# Supplementary material for: Victory Tax: A Holistic Income Tax System
Source: Entropy (Basel). 2021 Nov 11;23(11):1492. doi: 10.3390/e23111492 (PMC8624733; doi:10.3390/e23111492)
Supplement: Supplementary file 1 [file entropy-23-01492-s001.zip › bundle/SupplementalData.pdf]

SUPPLEMENTARY MATERIALS

Victory Tax: An Holistic Income Tax System

Donald J. Jacobs[1,2,*]

[1]Department of Physics and Optical Science, UNC Charlotte, Charlotte, NC 28223 USA.

[2]Affilitate faculty of the UNC Charlotte School of Data Science, Charlotte, NC 28223 USA.

*Corresponding Author Email: djacobs1@uncc.edu

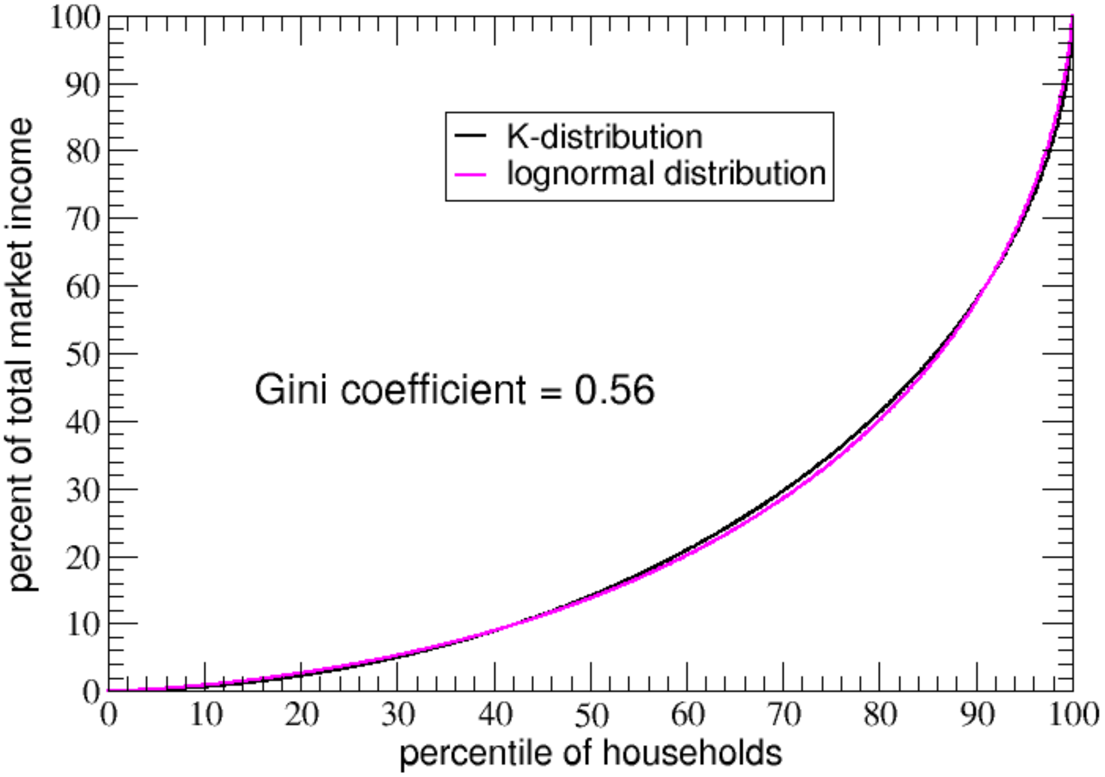

Figure S1: The Lorenz curves for the κ-distribution (economy A) and the lognormal distribution (economy B) are compared. Both models are fitted to the Internal Revenue Service tax data for 2003. Although the κ-distribution is a more accurate model, this graph shows that the Lorenz curves from both models are virtually the same, with Gini coefficients identical to the hundredths place. Note that percentile of households refers to a ranking from lowest income to highest income. This graph indicates that about 45% of all new income generated in 2003 went to the top 10% of households with the greatest income. In a proportionate system, when income is skewed the amount of tax paid will also be skewed. A flat tax would make it such that the top 10% of households with the greatest income would generate 45% of the collected tax revenue. A (regressive, progressive) tax system would mean that the top 10% of households would pay (less, more) than 45% of the total tax revenue collected.

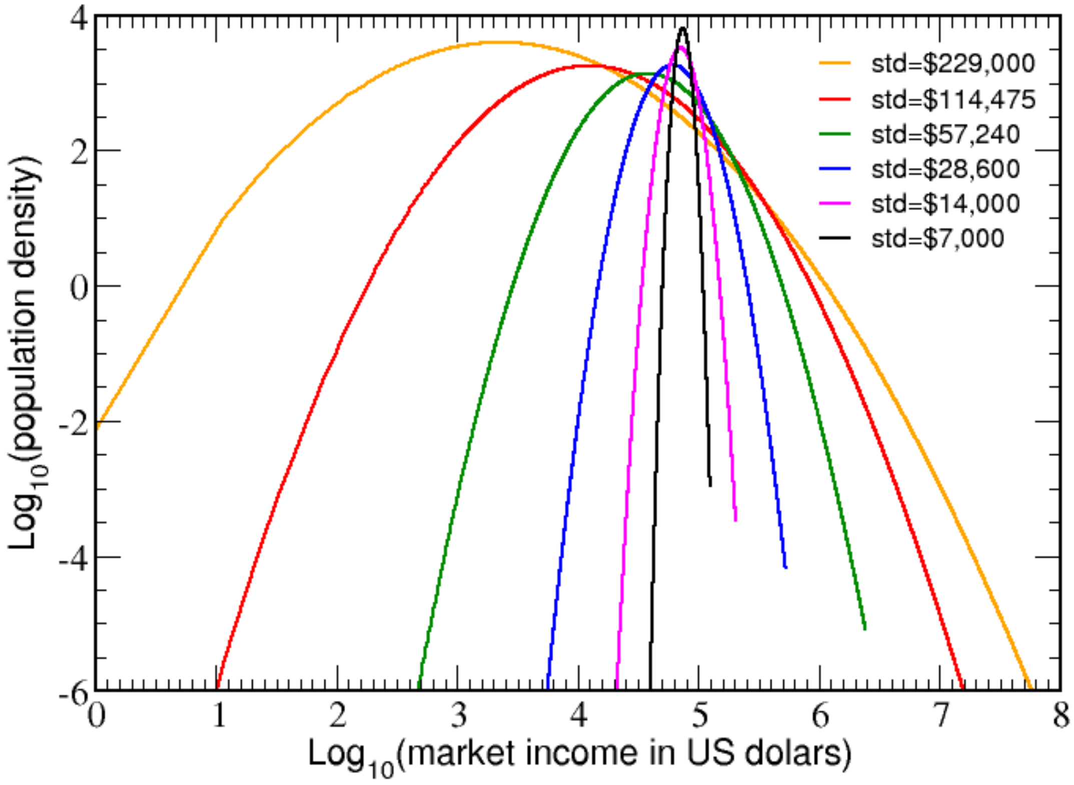

Figure S2: The lognormal distribution has two parameters to adjust, which is the mean income of a household, and the standard deviation of income about the mean. On a log10-log10 scale, six lognormal distributions are plotted, where the 2003 mean household income of $75,300 (adjusted to 2009 dollar values as a point of reference) is common among all cases shown. Differences among the distributions come from different standard deviations, listed in the legend, ranging from $7,000 to $299,000. When the standard deviation is small compared to the mean household income, the income for all households approaches the same value. In this case, it becomes rare to find households with much less or much greater incomes than the mean value. Conversely, standard deviations much larger than the mean household income allows a small percent of households to have more than three orders of magnitude more (or less) income than the population mean. These trends are visualized in this figure, where the plotted concave parabola-shaped lines are the most narrow when the standard deviation is small, and the curves broaden as standard deviations increase. The peak height in each curve corresponds to the most probable household income (or mode), which is always smaller than the mean. This skewness arises because a relatively small percent of households generate relatively very large amounts of income, making the mean greater than the median or mode. The reason for considering these six test economies is that the effect of income dispersion within a population on the amount of tax revenue collected, or on effective tax rates can be investigated explicitly.

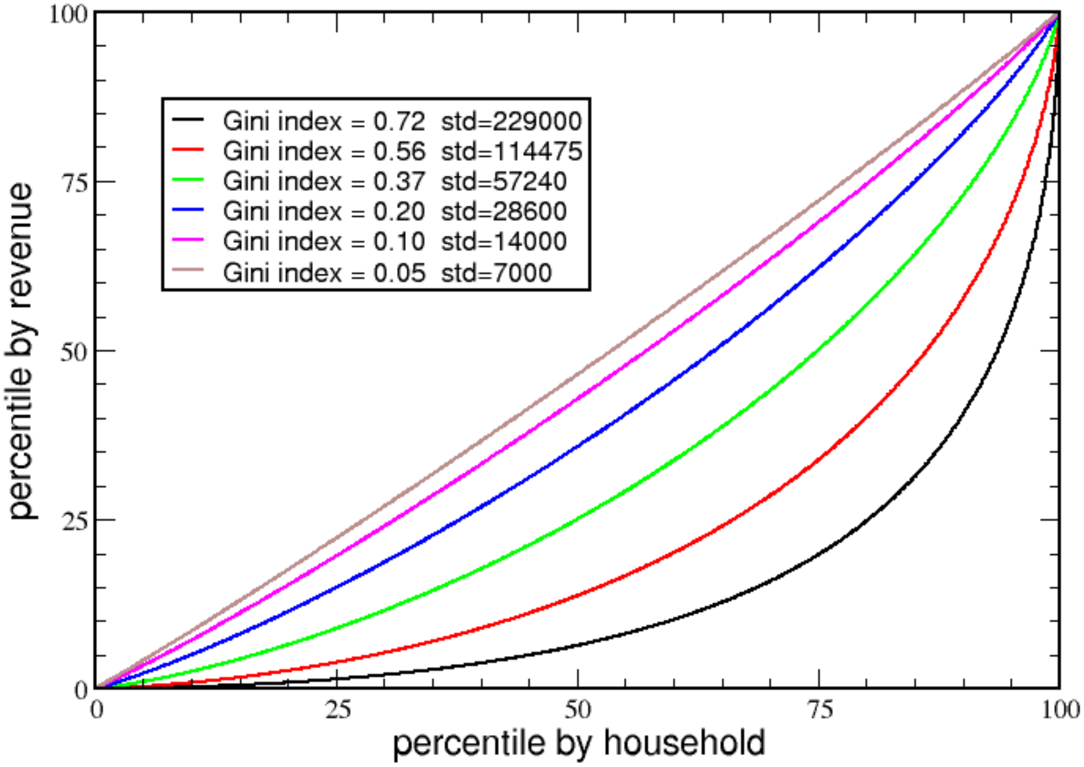

Figure S3: The Lorenz curves for each of the six test economies shown in Figure S2 using the lognormal distribution are plotted. The legend reports the Gini index and the standard deviation for each Lorenz curve. A standard deviation of $144,475 models the 2003 US economy well, and this distribution of income is called economy B in this work.

Figure S4A

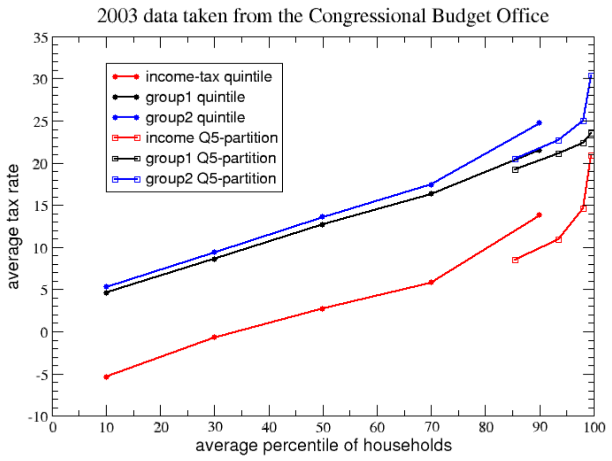

Figure S4B

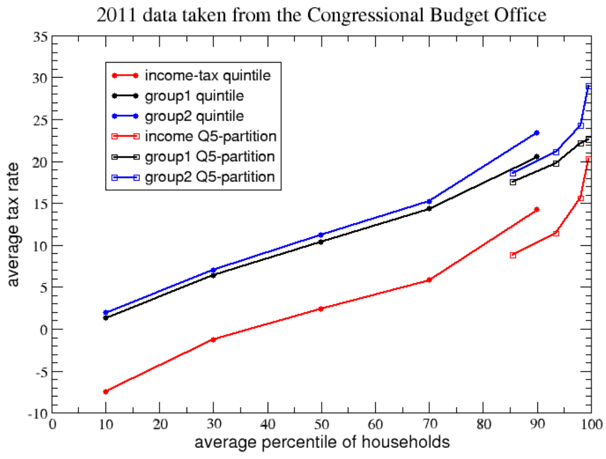

Figure S4: Average tax rate from the Congressional Budget Office (https://www.cbo.gov) based on household income federal tax data. Within quintiles the average tax rate is plotted against the average position of the quintile (solid circles). The fifth quintile is partitioned into percentile bins ranging from 81-90, 91-95, 96-99 and 99-100 where the average tax rate is plotted against the average position within each bin (open squares). Three types of tax rates are plotted. **(1)** The income tax rate (red lines); **(2)** the tax rate for *income plus payroll plus excise tax* called group1 (black lines), and **(3)** *group1 plus corporate tax associated with household investments* is called group2 (blue lines). Interestingly, the tax rates of all three categories for 2003 and 2011 exhibit similar characteristics despite disparate economic conditions, where 2003 marked the dawn of a booming economy while 2011 marked the turning point from a severe nationwide recession. This data shows that on average the US tax system is progressive. However, a progressive tax system does not allow an effective tax rate for household B with greater income than household A be less than the effective tax rate of household A. These cases frequently occur in US tax code due to various types of deductions or having different tax rates for different types of income. Note that in the CBO analysis, corporate tax is linked with personal income tax. In this work, the scenario that there is no corporate tax is assumed in order to establish a reference point from which direct comparisons of tax systems can be made.

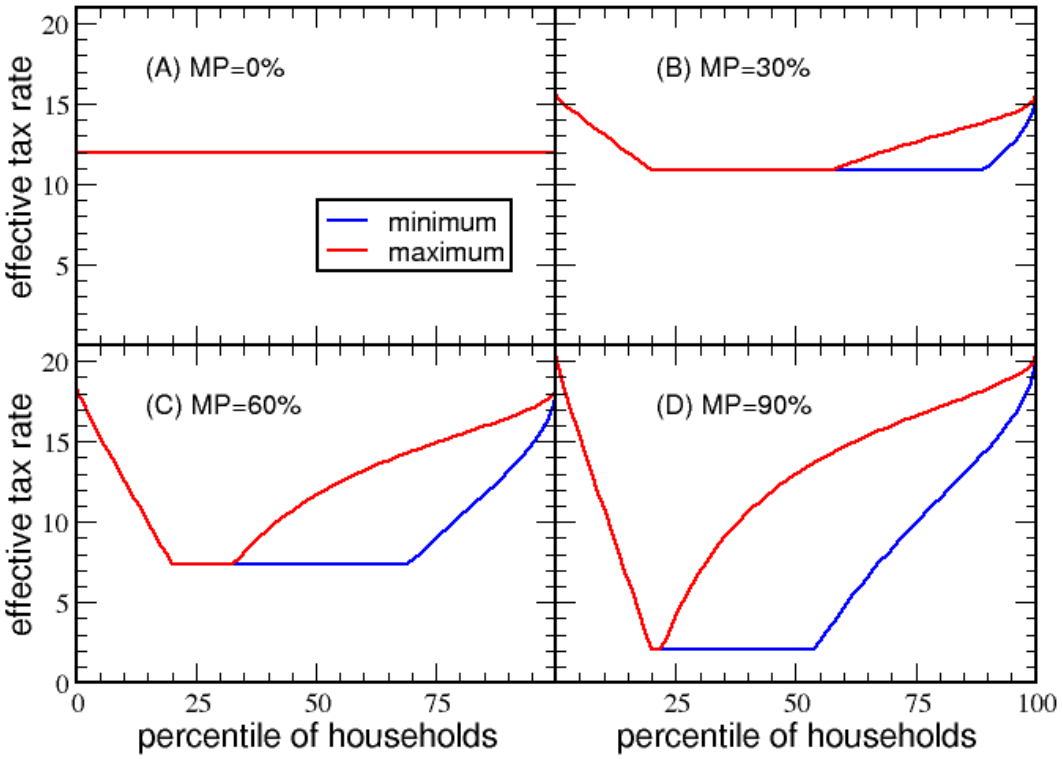

Figure S5: For economy B, the effective tax rates resulting from the victory tax system is shown for when the parameters {BD, MD} are set to BD=20% and MD=50%. A household that elects not to use any allowed itemized deductions will pay the greatest effective tax rate shown by the red line, whereas taking the maximum allowed itemized deductions yields the lowest possible effective tax rate shown by the blue line. The victory tax system sets the limits for the maximum and minimum effective tax rates. Until a household has enough self-generated after-tax income above the poverty line, the basic deduction exceeds the maximum allowed deductions possible. In this case the minimum and maximum deductions are equal, meaning the red line covers the blue line. The effective tax rates across households with varying income levels for economy A and economy B, both modeling the 2003 US economy, are very similar. This means the households with ultra-high income captured by economy A, but not in economy B, do not have a dramatic affect on the effective tax rate across the general population. Interestingly, it becomes clear that tax rates increase whenever ultra-high income households skew the mean income further away from the median. Since the victory tax system has a single tax rate for all households, independent of income level, no disproportionate tax burden is created across the population. Instead, all households become subjected to a higher tax rate as the dispersion in income across the population increases. Effective tax rates increase more for higher-income households when income dispersion across households increases (see Figure S6).

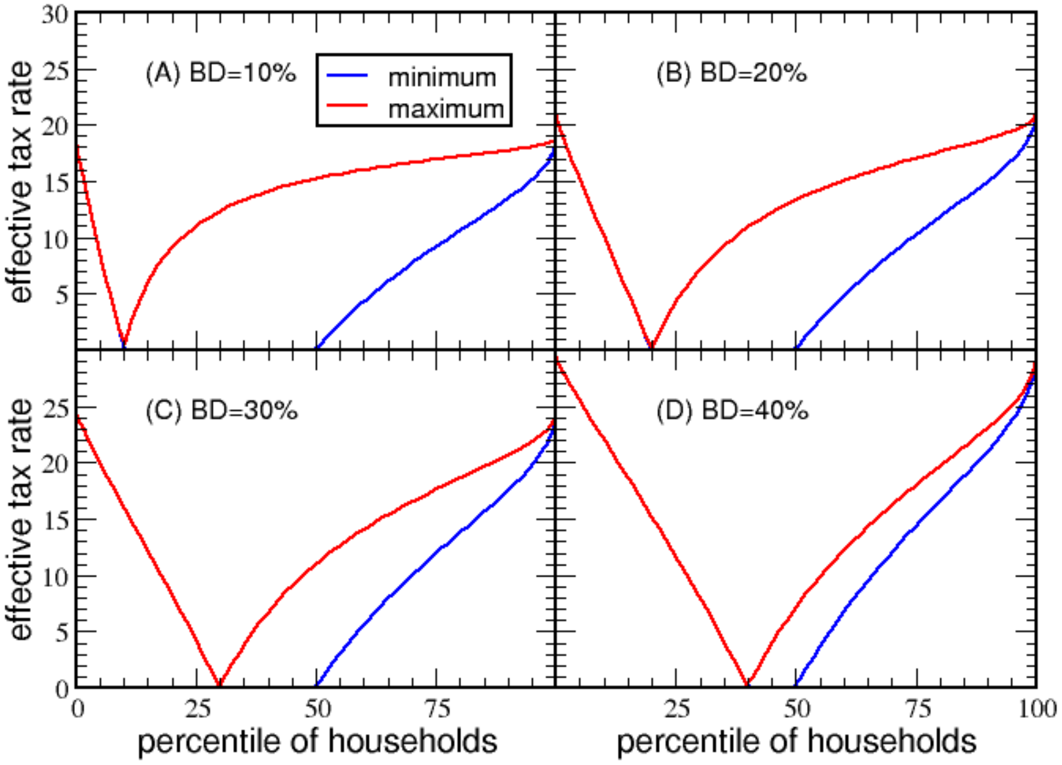

Figure S6: For economy B, the effective tax rates resulting from the victory tax system is shown for when the parameters {MD, MP} are set to MD=50% and MP=100%. Respective effective tax rates as a function of household percentile for economy B are slightly lower than for economy A. For example, effective tax rates differ between economies A and B by as much as a few tenths of a percent for low-income households and 1.4% for high-income households. Qualitatively, all trends are the same, as described next. The characteristic "V" shape occurs when no itemized deduction is taken, yielding the maximum possible effective tax rate, shown by the red line. Taking the maximum allowed itemized deductions yields the lowest possible effective tax rate shown by the blue line. Note that the basic deduction exceeds the maximum allowed deduction possible until the self-generated after-tax income of a household is above the poverty line. This is why the red line covers the blue line. Also the blue line indicates that (MD-BD)-percent of households can have a 0% effective tax rate, but the x-axis (black line) is covering the blue line. For example, in panel (A) 40% of households would have a 0% effective tax rate ranging from the 10 to 50 percentile of households. However, this case is possible only if the poverty line was under the 10% percentile. As the poverty line increases, the basic deduction (BD) must increase by definition to account for basic living expenses. Another example is shown in panel (D) where 10% of households can have a 0% effective tax, between the 40 to 50 percentiles of households. Households that enjoy a 0% effective tax will be above the poverty line, and must have enough income at their disposal to afford to make investments and capitalize on the maximum allowed itemized deductions. Hence, middle class households enjoy the lowest effective tax rates within a victory tax system. At some point the effective tax rate increases progressively for households with much larger net incomes, allowing capital loss deductions to be utilized to a greater degree.

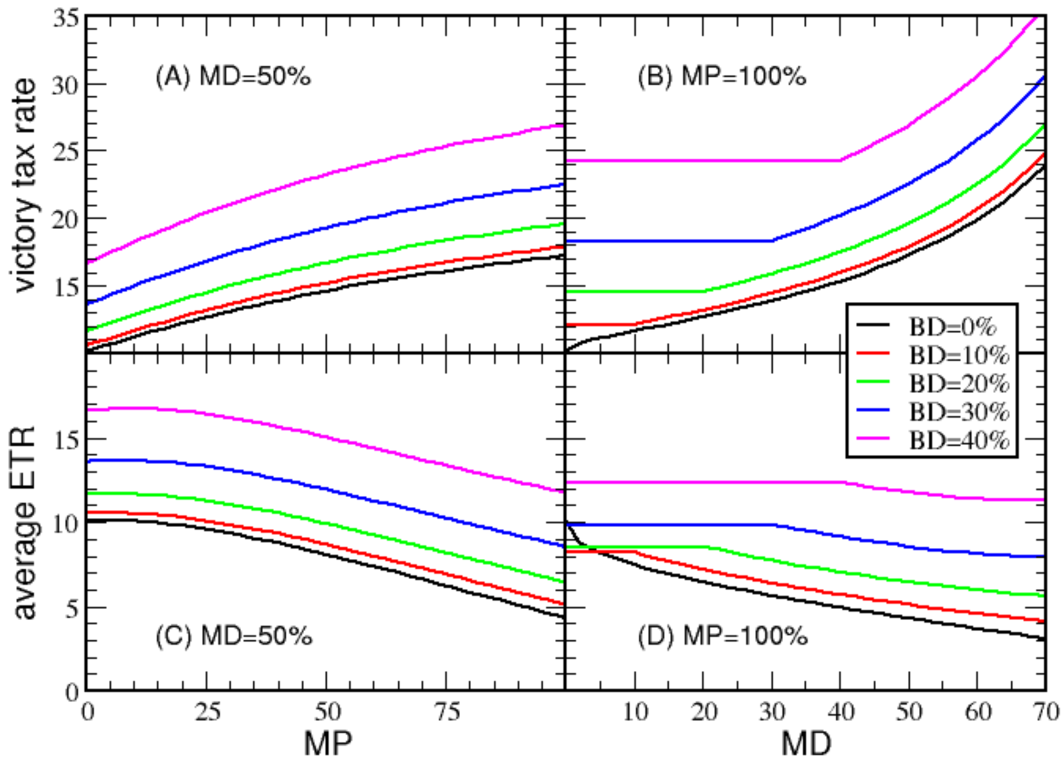

Figure S7: The victory tax rate and the average effective tax rate across all households for various parameter settings are shown for economy B. For panels (A) and (C) the MD parameter is set at MD=50% and the MP parameter is varied. For panels (B) and (D) the MP parameter is set at MP=100%, and the MD parameter is varied. For all panels, five different cases for the BD parameter is considered, each identified by a different colored line as specified in the legend that applies to all panels. Similar results are obtained for economies A and B, but differences are present. Namely, economy B has a lower victory tax rate, and a lower average effective tax rate than economy A. This shows that as greater extremes are present in the way income is distributed (more poor and more ultra-rich as modeled more accurately using model A), then tax rates increase. This effect will be investigated further using the six test economies described in Figures S2 and S3.

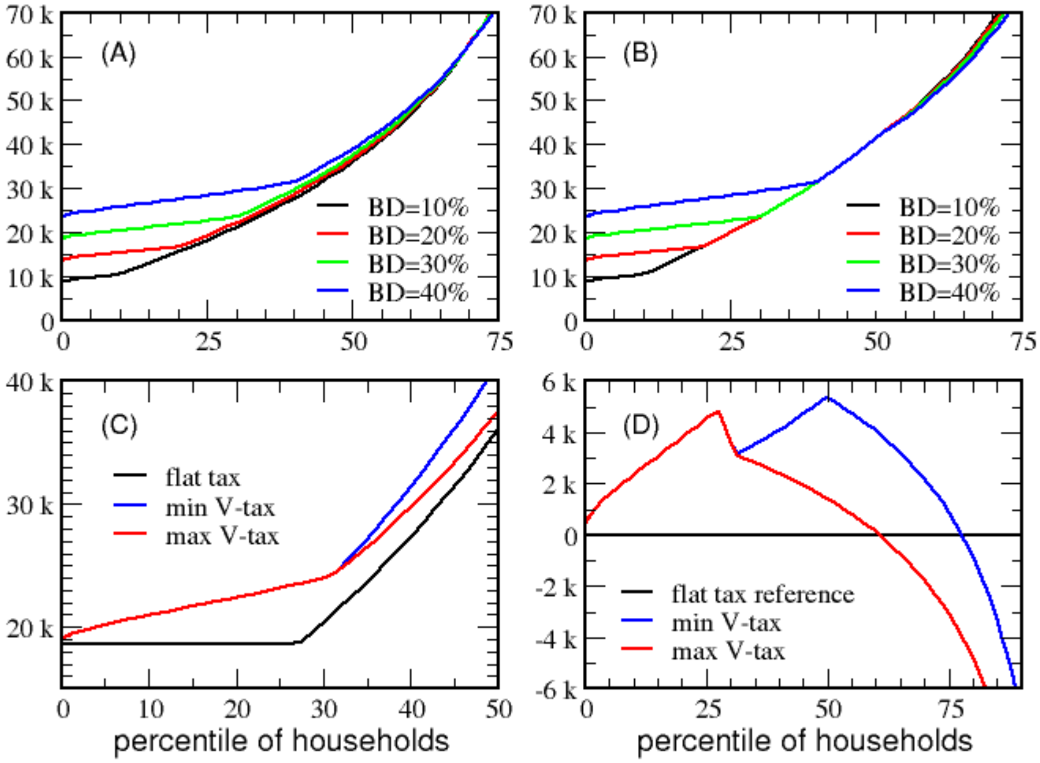

Figure S8: Results from the victory tax system for economy B when the MD parameter is set at MD=50% and the MP parameter is set at MP=100%. In panels (A) and (B) the minimum and maximum after-tax incomes are plotted respectively, up to the 75-percentile of households for basic deduction levels ranging from BD=10% to 40%. Shortly beyond the 75-percentile, the range on the y-axis becomes too large to see differences between the minimum and maximum after-tax incomes. Indeed, it is already difficult to see differences between panels (A) and (B). In panel (C) the case of BD=30% is plotted again, except the minimum and maximum after-tax incomes are plotted together up to the 50-percentile, and, after-tax income for a flat tax is shown as a black line corresponding to the special case of the victory tax system when MP=0%. The blue line shows the maximum after-tax income possible, achieved with a minimum effective tax rate by using all allowed itemized deductions. The red line shows the case when no itemized deductions are taken, and only the automatic basic deductions are applied. The red line covers the blue line up to the point that a household has self-generated after-tax income that exceeds the poverty line. Notice that a household that is fully supported by government transfer has an after-tax income equal to the poverty line, which is the y-intercept where the red, blue and black lines all intersect. Panel (D) shows the differences between the minimum and maximum after-tax incomes for the same case plotted in panel (C), but using the after-tax income from a flat tax as a reference line. Consequently, compared to a flat tax, one can readily see on the y-axis how much more or less after-tax income a household will have. From panel (D) it is clear that the victory tax system provides more after-tax income for the majority of households, but reduces after-tax income from households with the highest incomes.

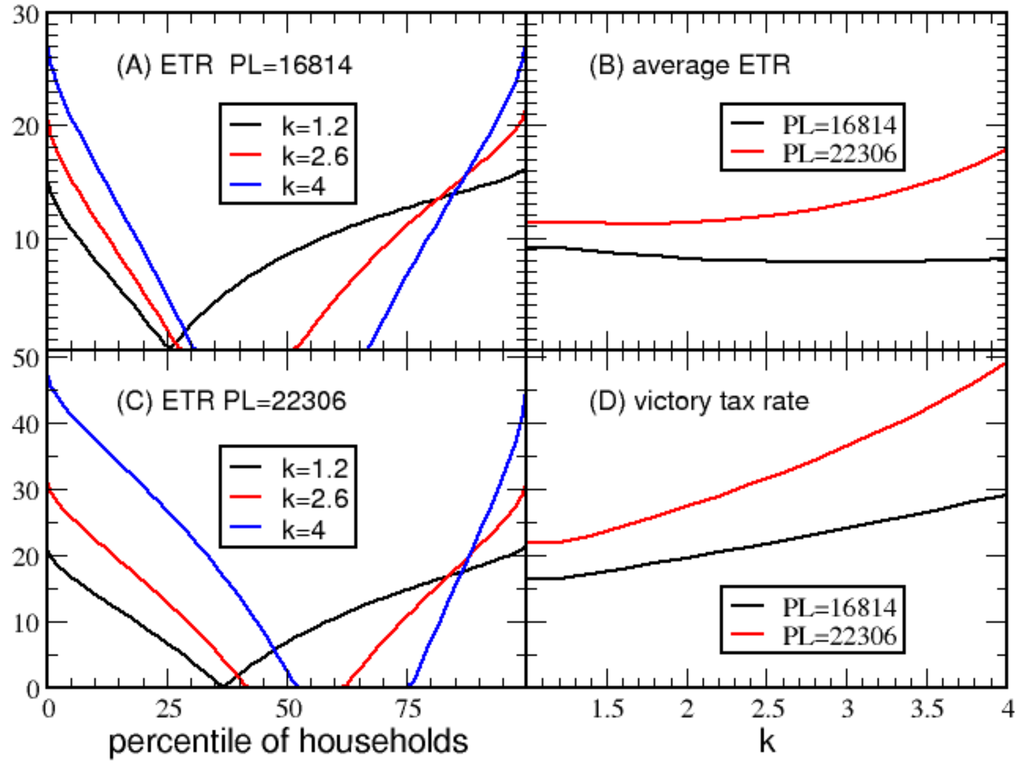

Figure S9: For economy B: Panels (A) and (C) plot effective tax rates as a function of percentile of households when the poverty line is at $16,814 and $22,306 respectively. A prototypical victory tax system sets the maximum deduction to be proportional to the poverty line, using the parameter k. By considering three different values for k, as specified in the legends, it is clear that the lowest effective tax rates (at 0% or near 0%) will be applied to households with different income levels, which can be controlled to some degree by the value of k. Also, as k increases there is more opportunity for households to achieve a 0% effective tax rate. To see this more clearly, as a function of k, panel (B) plots the average effective tax rate over all households, and panel (D) plots the victory tax rate. In both panels, two different values for the poverty line are considered. Although there is close quantitative agreement between the results from economy B and economy A, the tax rates for economy B are slightly lower as previously noted in Figure S6.

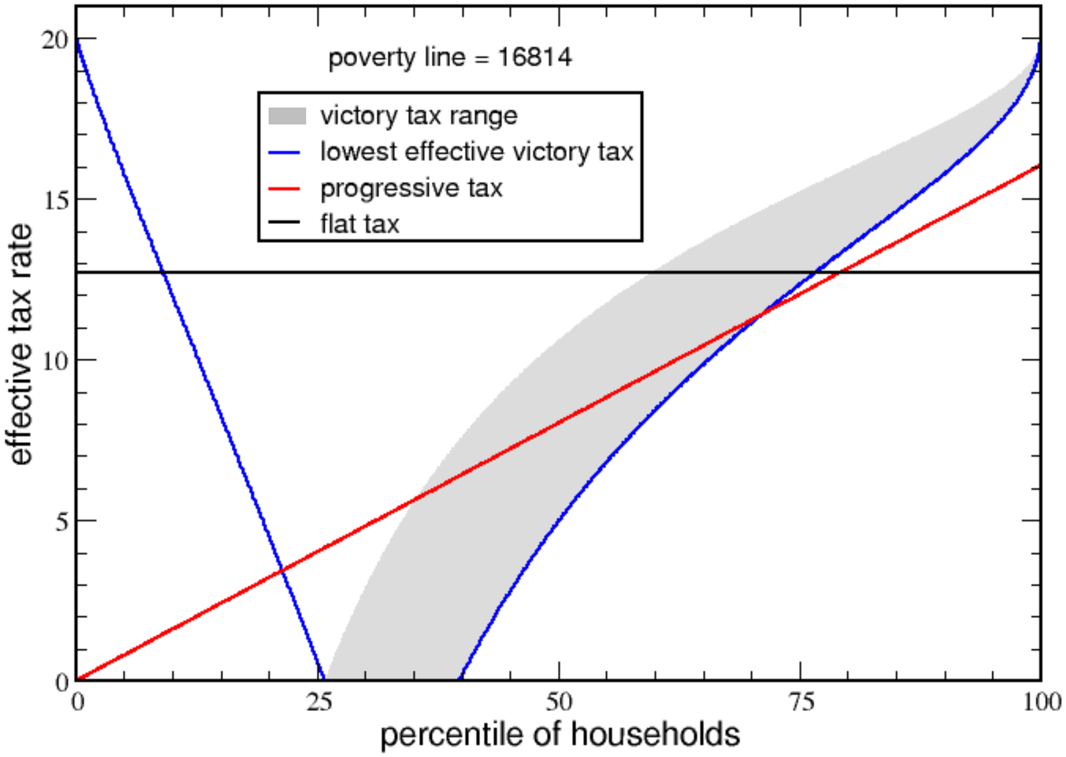

Figure S10: For a poverty line of $16,814 and economy A, the effective tax rates for flat, progressive and victory tax systems are compared. The progressive tax system considered here is "perfect", meaning that it is a linear function of percentile of household income, and has no deductions. As such, the flat tax and the progressive tax systems have no basic and itemized deductions. On the other hand, a victory tax provides households with basic and itemized deductions. For purpose of comparison, capital loss deductions are treated identically in all cases because it affects the definition of net income. Since itemized deductions are elective for households, it is possible for a household with an income in the 35-percentile, for example, to pay a 0% effective tax rate if the maximum allowed itemized deductions are taken. Alternatively, having no itemized deductions, this same household would be subjected to a 5.5% effective tax rate. As such, the grey area shown in the graph reflects the range of variability in effective tax rate that comes from the flexibility of itemized deductions. While it might seem that a household should always take the maximum allowed itemized deductions, it should be remembered that tax law would require certain allowed actions for these deductions, such as having a mortgage interest, investing in stocks, bonds or other forms of securities, possibly for higher education expenses, or possibly to encourage the use of new technologies that are beneficial to society in the long term, such as buying energy efficient devices that tend to be expensive in the short term. In addition, to cover a desired standard of living or various types of non-optional living expenses, a household may not have available funds to take advantage of all the allowed itemized deductions to maximum extent. It is worth pointing out here that the victory tax system defines limits in terms of mathematics, but detailed tax law that govern the allowed itemized deductions (and capital loss deductions) remains a matter of public policy.

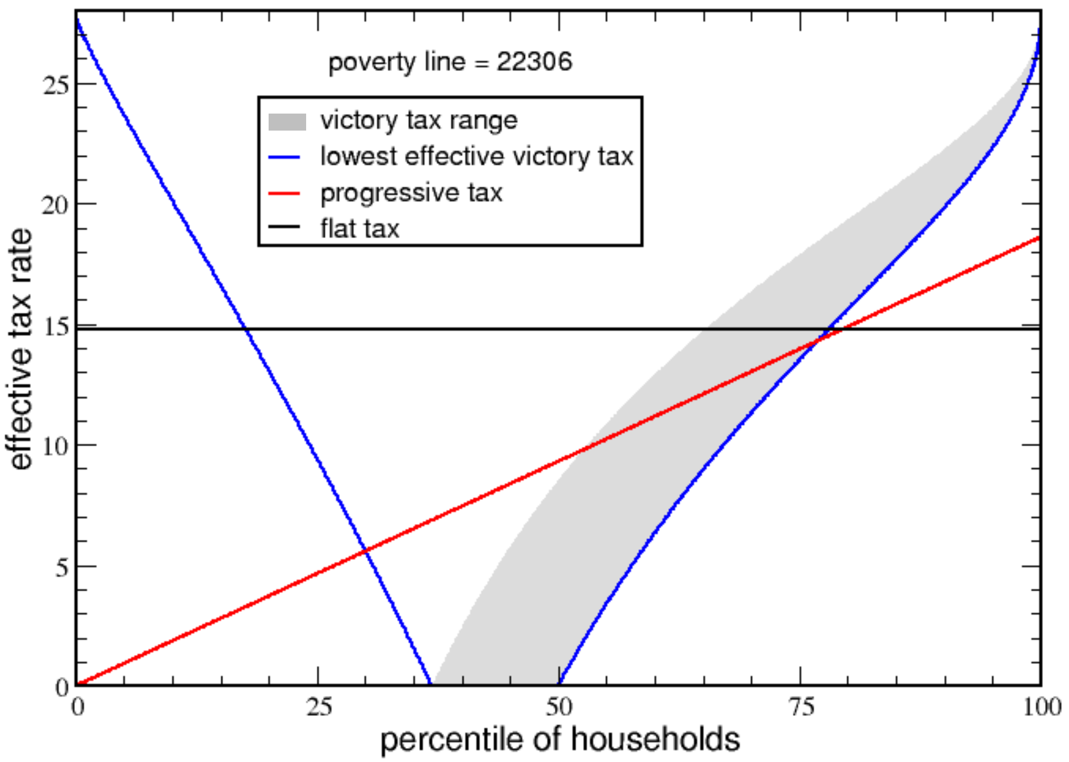

Figure S11: For a poverty line of $22,306 and economy A, the effective tax rates for flat, progressive and victory tax systems are compared. It is interesting to compare this case with that presented in Figure S10 (having a poverty line of $16,418). As the poverty line increases, the percentile range where households can have a 0% effective tax rate increases. To pay for government "generosity" to abolish poverty, it is clear that tax rates must increase, regardless of the tax system. Comparing this figure with Figure S4, suggests the highest income households in a victory tax would pay an effective tax rate just under 28%, while in year 2003, the US tax system put the effective tax rate after government transfer at about 14% and before government transfer at about 25%. Given that the numbers in this report are based on having a balanced budget, the victory tax rate of 28% need not be lower because it does much more public good than what the US tax system did in 2003 (or any year there was a deficit or poverty). Namely, the advantage of an overall higher tax rate is that the system eliminates deficits and poverty, and it also fuels the economy by supporting a strong middle class that is critically important for maintaining long-term stability of a government. Furthermore, for a consumer-based economy, the majority of the gross domestic product derives from the spending power of the middle class. While a 28% victory tax rate does not impose excessive tax burden, the simplest way to reduce the value is for a stronger middle class to emerge. This is likely given that the middle class has more resources to invest and for the lower-income houses to move into the middle class. For example, the average effective tax rate across the population is only about 12%, with the vast majority of the population having a low ETR. The middle class enjoys the lowest effective tax rates that will be 0% for up to 15% percent of households.

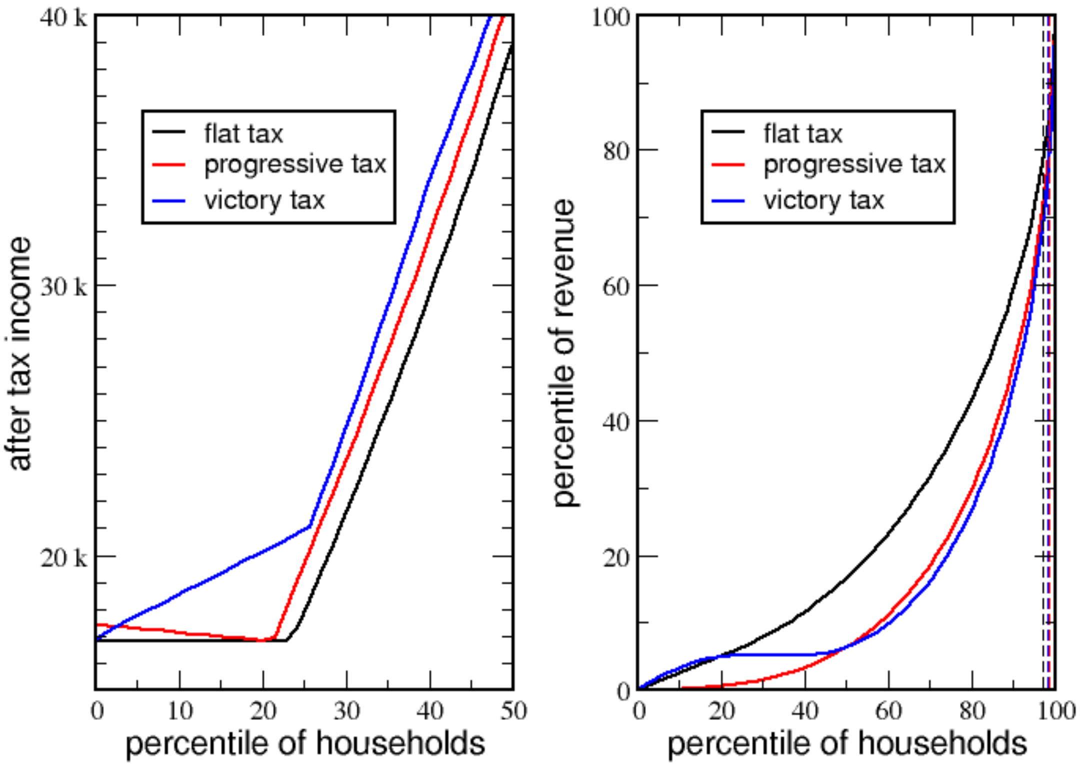

Figure S12: For economy A, the left panel plots after-tax income up to the 50-percentile for a flat, progressive and victory tax system. Each case yields the same tax revenue, and has the same poverty line, which is set here to be at $16,814. Compared to a $22,306 poverty line, the after-tax income is greatly diminished for households within the 30-percentile. Due to relative scale, small differences in after-tax income can greatly impact living standards in low-income households. Corresponding Lorenz curves are plotted in the right panel. Qualitative features for a poverty line of $16,814 are the same as that for a poverty line of $22,306. The vertical dashed lines of corresponding color mark a dividing percentile of households, where tax revenue from all households above this marked percentile is used to pay for all government transfers. To the nearest tenths place, these marked percentiles are 97.0%, 98.7% and 98.1% for the flat, progressive and victory tax systems respectively. If households below the poverty line are given the same level of assistance, then the tax systems having the lowest tax rate on the highest income households will yield the lowest marked percentile. For example, the flat tax has the lowest tax rate among the three tax systems compared, suggesting a greater number of high income households are needed to contribute to government transfer. However, as the left panel indicates, each tax system provides different levels of support to households below the poverty line. Interestingly, for the three tax systems compared (flat, progressive, victory) the percent of total tax revenue that goes toward government transfers is roughly (23%, 19%, 26%). For the victory tax system, the top 2% of high-income households pay approximately 26% of the total tax revenue, which is redistributed as government transfer to eradicate poverty. As a point of reference, this same set of households accumulate about 18% of the total income within the economy, which reflects the progressive nature of the victory tax system at high incomes. In contrast, for a flat tax system, the top 3% of high-income households accumulates about 23.1% of the total income, and pays 23.9% of the total tax, which is very close to the result of a perfect flat tax, which does not allow any redistribution of income whatsoever.
