# Supplementary material for: Victory Tax: A Holistic Income Tax System
Source: Entropy (Basel). 2021 Nov 11;23(11):1492. doi: 10.3390/e23111492 (PMC8624733; doi:10.3390/e23111492)
Supplement: Supplementary file 1 [file entropy-23-01492-s001.zip › bundle/results_to_give_away/data4VTpaper/TaxTableExample4.pdf]

Line #	Question/Percentile of household	0	10	20	30	40	50
1	Government support	$30,871	$22,383	$14,540	$6,228	$0	$0
2	Earnings income	$0	$8,488	$16,326	$24,603	$33,705	$44,064
3	Other income (*from worksheet*)	$0	$0	$5	$40	$173	$558
4	Total deductible income (*add lines 2 and 3*)	$0	$8,488	$16,331	$24,643	$33,878	$44,622
5	Basic deduction (*from table*)	$30,871	$30,871	$30,871	$30,871	$30,871	$30,871
6	Itemized deductions *(from worksheet)*	$0	$0	$0	$0	$241	$1,719
7	Total deductions (*add lines 5 and 6*)	$30,871	$30,871	$30,871	$30,871	$31,112	$32,590
8	Reduced income (*subtract line 7 from line 4*)	($30,871)	($22,383)	($14,540)	($6,228)	$2,766	$12,032
9	Adjusted income (*the greater of line 8 or 0*)	$0	$0	$0	$0	$2,766	$12,032
10	Taxable income (*add lines 1 and 9*)	$30,871	$22,383	$14,540	$6,228	$2,766	$12,032
11	Tax owned (*multiply line 10 by 0.277443*)	$8,565	$6,210	$4,034	$1,728	$768	$3,338
	effective tax rate	27.7%	20.1%	13.1%	5.6%	2.3%	7.5%

Line #	Question/Percentile of household	60	70	80	90	95	99
1	Government support	$0	$0	$0	$0	$0	$0
2	Earnings income	$56,289	$71,677	$93,501	$135,429	$189,913	$411,893
3	Other income (*from worksheet*)	$1,498	$3,616	$8,343	$20,455	$36,958	$97,951
4	Total deductible income (*add lines 2 and 3*)	$57,787	$75,293	$101,844	$155,884	$226,871	$509,844
5	Basic deduction (*from table*)	$30,871	$30,871	$30,871	$30,871	$30,871	$30,871
6	Itemized deductions *(from worksheet)*	$4,845	$10,883	$13,741	$13,741	$13,741	$13,741
7	Total deductions (*add lines 5 and 6*)	$35,716	$41,754	$44,612	$44,612	$44,612	$44,612
8	Reduced income (*subtract line 7 from line 4*)	$22,071	$33,539	$57,232	$111,272	$182,259	$465,232
9	Adjusted income (*the greater of line 8 or 0*)	$22,071	$33,539	$57,232	$111,272	$182,259	$465,232
10	Taxable income (*add lines 1 and 9*)	$22,071	$33,539	$57,232	$111,272	$182,259	$465,232
11	Tax owned (*multiply line 10 by 0.277443*)	$6,123	$9,305	$15,879	$30,872	$50,566	$129,075
	effective tax rate	10.6%	12.4%	15.6%	19.8%	22.3%	25.3%
