# Supplementary material for: Victory Tax: A Holistic Income Tax System
Source: Entropy (Basel). 2021 Nov 11;23(11):1492. doi: 10.3390/e23111492 (PMC8624733; doi:10.3390/e23111492)
Supplement: Supplementary file 1 [file entropy-23-01492-s001.zip › bundle/results_to_give_away/supporting_gov_data/government transfer payment.pdf]

May 6, 2003

Transfer Payments

In personal income, transfer payments are income payments to persons for whom no current services are performed. They are payments by government and business to individuals and nonprofit institutions.[1]

Transfer payments accounted for more than 13 percent of total personal income at the national level in 2001 (table F).

Estimates are prepared for approximately 50 subcomponents of transfer payments.[2] For organizational convenience, the subcomponents are classified by source--government or business--and by recipient--individuals or nonprofit institutions. In this discussion, transfer payments consists of three major components--government payments to individuals, government and business payments to nonprofit institutions, and business payments to individuals.

At the county level, approximately 75 percent of the estimates of transfer payments are derived from data for the payments. The remaining 25 percent are allocations of the State estimates in proportion either to data that are related to the components or to the most relevant population series.

This section is organized according to the order of the presentation of the components and subcomponents in table F. Each estimated item is briefly defined, and the preparation of the county estimates is described.

---

1. Transfer payments from the rest of the world are netted against similar payments to the rest of the world, and the net payments, called "personal transfer payments to the rest of the world (net)," are entered in the national income and products accounts as part of personal outlays.

2. The estimates of transfer payments in subcomponent detail for 1969-2001 for States, counties, and metropolitan areas are available in table CA35 on this disc.

Government Payments to Individuals

Retirement and disability insurance benefit payments

Old-age, survivors, and disability insurance (OASDI) benefits.--These benefits, popularly known as social security, consist mainly of monthly benefits received by retired and disabled workers, dependents, and survivors and lump-sum payments received by survivors.

The State estimates are based on annual tabulations of payments from the Social Security Administration (SSA). The county estimates are based on SSA tabulations of the amount of monthly benefits paid to those in current-payment status on December 31 by county of residence of the beneficiaries.

Railroad retirement and disability benefits.--These benefits are received by retired and disabled railroad employees and their survivors under the Federal program of retirement insurance for railroad employees, who are not covered by OASDI.

The State and county estimates are based on tabulations of the benefits disbursed by the Railroad Retirement Board.

Workers' compensation benefits.--These benefits consist of the payments that are received by individuals with employment-related injuries and illnesses from publicly administered workers' compensation insurance from both the Federal and State governments.

The State estimates of the payments received under the Federal program, which covers only Federal civilian employees, are based on payments data from the Department of Labor. In the absence of data for counties, the State estimates are allocated to counties in proportion to the estimates of Federal civilian wages and salaries, which are adjusted to a place-of-residence basis.

Benefit payments to both public and private employees from State-administered workers' compensation funds consist of the payments received under exclusively State-administered workers' compensation insurance programs, the payments received under State-administered

insurance programs that compete with private insurance programs, and the payments received under State programs for second-injury funds.[3]

The State estimates of these benefits are derived from data for the payments from State workers' compensation funds by the State of work from the Census Bureau's annual State Government Finances. These data are adjusted to a place-of-residence basis by BEA. In the absence of payments data for counties, the State estimates are allocated to counties by the sum of the estimates of wages and salaries for private employees and State and local government employees, which are adjusted to a place-of-residence basis.

Other government retirement and disability insurance payments.--These payments consist of the payments of temporary disability benefits, the payments of black lung benefits, and the payments of benefits from the Pension Benefit Guaranty Corporation.

Temporary disability benefits are the benefits received by workers who are unemployed because of nonoccupational illnesses or injuries. These benefits are from State-administered programs only in California, New Jersey, New York, and Rhode Island.

The State estimates are allocated to counties in proportion to the civilian population.

Black lung benefits are the benefits received by the coal miners who are totally disabled by black lung disease (pneumoconiosis) and by the eligible survivors of miners whose deaths were caused by the disease. Individuals whose eligibility was established before July 1973 receive their benefits from the SSA; those whose eligibility was established since June 1973 receive benefits from the Department of Labor.

The State estimates of payments are based on State data from both agencies. However, only the SSA provides county data. The State estimates are allocated to counties in proportion to annual SSA tabulations of benefit payments for the month of June.

---

3. Second-injury funds underwrite the risk of a subsequent work-related injury to an already disabled worker. Therefore, the liability of the employer of a disabled worker is limited to the liability for the impairment resulting from the injury sustained during the present employment. The difference between the compensation for the full impairment and the employer's liability is paid out of the second-injury fund.

Pension Benefit Guaranty benefits are paid by the revolving fund of the Pension Benefit Guaranty Corporation (PBGC) to individuals whose PBGC-insured pensions cannot be paid by the private pension funds that are liable for the benefits.

The national estimate is allocated to States and counties in proportion to OASDI benefits, which are assumed to reflect the geographic distribution of the retired population.

Medical benefits

Medicare benefits.--These benefits are Federal Government payments made through intermediaries to beneficiaries for the care provided to individuals under the provisions of the medicare program.

The State estimates of the payments under the medicare provisions for hospital insurance and supplementary medical insurance are based on adjusted fiscal year data for the amounts that are paid as reimbursement for hospital and medical expenses as reported by the Centers for Medicare and Medicaid Services (CMS).

The State estimates are allocated to counties by the dollar amounts that are paid as reimbursement for hospital and medical expenses, classified geographically according to the residence of the beneficiaries. These data are drawn from the Adjusted Average Per Capita Cost master file from CMS. Because these data are no longer being compiled, the county estimates for 1996-2001 are extrapolated from the estimates for 1995 by the change in medicare enrollment from CMS.

Medical vendor payments.--These medical benefits are received by low-income individuals; the benefits are called vendor payments because they are measured as the payments to the vendors of the medical services. These payments consist mainly of the payments made through intermediaries to the vendors for care provided to individuals under the federally assisted, State-administered medicaid program and State children's health insurance program (SCHIP), but these payments also include payments made under the general assistance medical programs of State and local governments.

The State estimates of the payments are based on payments data from CMS.

For about two-thirds of the States, the county estimates of payments made under both medicaid and the general assistance medical programs are based on medicaid payments data from the State departments. See table G for a list of these States and for the most current year for which the payments data are available for each State. For a year or years for which the data are not available, the available data for the most recent year are used to prepare the county estimates. For the States for which payments data are not available by county, the State estimates of all payments are allocated to counties in proportion to the payments made under the aid to families with dependent children program.

The county estimates of payments made under SCHIP are based on enrollment data from the various state departments of social services. For counties in States that do not provide these data, the state estimates are allocated in proportion to the estimate of people age 0-17 in poverty from the Census Bureau's Small Area Income and Poverty Estimates.

Military medical insurance benefits.--These benefits are vendor payments made under the TriCare Management Program, formerly called the Civilian Health and Medical Plan of the Uniformed Services program, for the medical care of dependents of active duty military personnel and of retired military personnel and their dependents at nonmilitary medical facilities.

The State estimates are based on payments data from the Department of Defense.

County data for these payments are unavailable. The State estimates are allocated to counties by military retirement payments data for September that are provided each year by the Department of Defense.

Income maintenance benefits

Supplemental security income (SSI) benefits.--These benefits consist of payments received by the aged, blind, and disabled from both the Federal and State governments.

The State estimates are based on SSA tabulations of annual disbursements for two categories of SSI benefits: Basic Federal payments and supplemental State payments. The county estimates of the combined categories are based on payments data reported by the SSA.

Family assistance.--Formerly, this assistance was provided through the Federally-aided, State-administered Aid to Families with Dependent Children (AFDC) and emergency assistance programs. In 1997 they were phased out and replaced by the Temporary Assistance to Needy Families (TANF) program.

The State estimates are based on unpublished quarterly data for these payments from the SSA. The county estimates are based on payments data from the various State departments of social services.

Food stamps.--These benefits are measured as the value of the food stamps issued to qualifying low-income individuals in order to supplement their ability to purchase food. Eligibility is determined by the State authorities' interpretation of Federal regulations; the U.S. Department of Agriculture (USDA) pays the cost of the stamps.

The State and county estimates are based on county tabulations of the value of the distributed stamps from the Department of Agriculture.

Other income maintenance payments.--These payments consist of general assistance benefits, foster care and adoption assistance payments, earned income tax credits, energy assistance benefits, and the value of vouchers issued under the Women, Infants and Children (WIC) program.

General assistance benefits are the payments received from State and local governments by low-income individuals and families who do not qualify for help under federally supported programs.[4]

The State and county estimates are based on payments data from the various State departments of social services.

4. The Federal Government neither funds nor regulates these programs.

Foster care and adoption assistance payments are received from State governments by families and institutions that care for foster children and by families that adopt children. These payments are made under state programs, some of which are federally aided.

The State estimates are based on data from the Administration for Children and Families (ACF) on the Federal grants adjusted to reflect the fund matching percentage that is required of each State. The State estimates were allocated to counties in proportion to the civilian population.

Earned income tax credits are Federal income tax refunds to low-income workers, mainly those with minor children. Eligibility for the tax credits is determined by the size of the adjusted gross income, or the earned income, and by certain household characteristics. Only a portion of this Federal income tax credit is counted as a transfer payment; this portion is the excess of the tax credit over the tax liability.

The State estimates are derived from tabulations of the amount of payments disbursed to the residents of each State from the Internal Revenue Service. Because county data are unavailable, the State estimates are allocated to counties in proportion to a series that was derived from data on earnings of families in poverty status from the 1990 Census of Population.

Energy assistance benefits consist of both cash payments received by needy households and vendor payments made to suppliers to help defray the cost of home heating, cooling, and weatherization under the federally funded and State-administered energy assistance programs.

The State estimates are based on payments data published by the SSA. The estimates for counties in most States are based on payments data from the various State departments of social services. For counties in States that do not provide these data, the State estimates are allocated to counties in proportion to SSI enrollment.

The **Women, Infants and Children (WIC)** program is fully funded by the USDA Food and Nutrition Service, operating mainly through State agencies. The transfer payments under the

program take the form of vouchers issued to low-income women who are pregnant or who have young children; the vouchers are used to purchase supplemental nutritious foods.

The State estimates are based on direct data provided by the Food and Nutrition Service. State estimates were allocated to counties in proportion to the estimates of AFDC and TANF benefits.

Unemployment insurance benefits

State unemployment compensation.--These benefits consist mainly of the payments received by individuals under State-administered unemployment insurance (UI) programs, but they include the special benefits authorized by Federal legislation for periods of high unemployment.[5] The provisions that govern the eligibility, timing, and amount of benefit payments vary among the States, but the provisions that govern the coverage and financing are uniform nationally.

The State estimates are based on payments data from the Department of Labor. The State estimates are allocated to counties by the payments data reported by the State employment security agencies (ESA's). Most of the data are reported by county. However, some of the data are reported by local district office; these data are allocated to the counties in the jurisdiction of the local district office in proportion to the estimates of the annual average number of unemployed individuals from the Bureau of Labor Statistics (BLS). When the availability of the county data is delayed, preliminary county estimates for a year are prepared and then revised the following year to incorporate the delayed county data. The preliminary estimates for a year are extrapolated from the estimates for the preceding year by the change in the BLS estimates.

5. The program for Federal civilian employees and that for veterans are administered by the States, but the benefits are classified in other subcomponents of unemployment insurance benefits.

<u>Unemployment compensation of Federal civilian employees</u>.--The UI program for Federal employees is a Federal program administered by the State ESA's acting as agents for the U.S. Government.

The State estimates are based on State data on unemployment compensation paid to Federal employees; these data are provided by the ESA's in all States. In about half of the States, the State estimates are allocated to the counties by county data or by local-district-office data; the allocation procedure that is used is the same as that used for the estimates of State unemployment compensation. For the remaining States, the county allocators are residence-adjusted estimates of Federal civilian wages and salaries.

<u>Unemployment compensation of railroad employees</u>.--These benefits are the payments that are received by railroad workers who are unemployed because of sickness or because work is unavailable. This UI program is administered by the Railroad Retirement Board (RRB) under a Federal formula that is applicable throughout the Nation.

The State and county estimates are based on data for these payments from the RRB.

<u>Unemployment compensation of veterans</u>.--These benefits are received by unemployed veterans who have recently separated from military service and who are not eligible for military retirement benefits.

The State estimates are based on payments data from the ESA's. For about half of the States, the State estimates are allocated to counties by county data or by local-district-office data from the ESA's; the allocation procedure that is used is the same as that used for the estimates of State unemployment compensation. For the remaining States, the county allocator is the population of veterans from the 1990 Census of population.

<u>Trade adjustment allowances</u>.--These benefits are the payments received by workers who are unemployed because of the adverse economic effects of international trade arrangements.

The State and county estimates are based on calendar year data for these payments that are tabulated by "petition" (location of plant) from the Department of Labor, which administers

the program. The estimates are residence adjusted by BEA to approximate a geographic distribution based on the place of receipt of the benefits.

Veterans benefit payments

Veterans pension and disability benefits.--These benefits consist primarily of the payments that are received by veterans with service-connected disabilities and by the survivors of military personnel who died of service-connected causes. In addition, these benefits are received by war veterans who are 65 years old or older, who have nonservice-connected disabilities, who are permanently and totally disabled, and who meet specified income requirements.

The State and county estimates are based on the data for these payments from the Department of Veterans Affairs (DVA).

Veterans readjustment payments.--These benefits are the payments of the allowances for tuition and other educational costs that are received by veterans and by the spouses and the children of disabled and deceased veterans; and for automobiles, conveyances, and specially adapted housing for disabled veterans.

The State and county estimates are based on data for these payments from the DVA.

Veterans life insurance benefits.--These benefits are the claims received by the beneficiaries and the dividends received by the policyholders from the five veterans life insurance programs administered by the DVA.

The State and county estimates are based on data for these payments from the DVA.

Other veterans benefits.--These benefits consist of the State and local government payments of assistance to indigent veterans, and the State and local government payments of bonuses to veterans.

The State estimates of the Federal Government payments are based on data for these payments from the DVA. The State estimates are allocated to counties in proportion to the population of veterans.

The State estimates of the State and local government payments of assistance and of bonuses are based on fiscal year data from the Census Bureau's annual State Government Finances. The State estimates are allocated to counties in proportion to the number of the veterans of the post-Korean-War period.

Federal education and training benefits

Federal fellowship benefits.--The benefits are the payments that are received by the recipients of Federal fellowships; these payments are a small portion of the total fellowship.[6]

These payments are estimated in three subcomponents: The payments to outstanding science students who receive National Science Foundation (NSF) grants, the subsistence payments to the cadets at the six State maritime academies, and the payments for all other Federal fellowships.

The State and county estimates of the payments to the recipients of NSF grants are based on annual NSF tabulations of the number of students receiving fellowships at each institution.

The State and county estimates of the subsistence payments to the cadets are based on payments data for each academy. The amount of the payment is assigned to the State and county in which each academy is located.

Because of the lack of pertinent data, the national estimates of the payments to the recipients of all other Federal fellowships are allocated to States and counties in proportion to the civilian population.

6. The large portion of a Federal fellowship is paid to the school that the recipient attends. This payment is classified as a transfer payment to a nonprofit institution if the school is privately administered, or it is classified as a government grant-in-aid if the school is publicly administered.

<u>Interest payments on guaranteed student loans</u>.--These payments are made by the Department of Education to commercial lending institutions on behalf of the individuals who receive low-interest, deferred-payment loans from these institutions in order to pay the expenses of higher education.

The national estimate is allocated to States in proportion to the number of individuals enrolled in institutions of higher education from the Department of Education. The allocator for the county estimates is the civilian population.

<u>Higher education student assistance</u>.--This assistance is the Federal payments, called Pell Grants, to students with low incomes for an undergraduate education.

The State and county estimates are based on tabulations of annual data for these payments by the county of the educational institution from the Department of Education.

<u>Job Corps benefits</u>.--These benefits are primarily the allowances for living expenses received by economically disadvantaged individuals who are between the ages of 16 and 21 and who are enrolled in the designated vocational and educational training programs. These benefits also include the adjustment allowances received by trainees upon the successful completion of their training.

The State estimates are based on calendar year tabulations of the amount of allowances and allotments disbursed to the enrollees; the tabulations are from the Employment and Training Administration of the Department of Labor. The State estimates are allocated to counties in proportion to the civilian population.

Other government payments to individuals

<u>Compensation of survivors of public safety officers</u>.--These benefits are payments to the survivors of State and local government employees, such as police officers and fire fighters, who are killed in the line of duty; the payments are made under a Federal program. In 1988, the

payment was $100,000. Since 1988, it has been $100,000 plus an allowance for the increase in consumer prices.

The national estimate is allocated to States by the tabulations of the number of claims by State from the Department of Justice. The county allocator is the number of claims by city from the same tabulations.

Compensation of victims of crime.--This compensation consists of payments to crime victims and to vendors on behalf of crime victims.

The national estimate of total payments is allocated to States in proportion to information assembled by the Crime Victims Board of the New York State Executive Department. The county allocator is the geographic distribution of the civilian population.

Alaska Permanent Fund benefits.--These benefits are the disbursements of investment income to the residents of Alaska from the Alaska Permanent Fund. The fund, which is derived from oil revenues, pays a portion of its net investment income to every resident.

The State estimate is the amount that is paid and that is reported by the State. The State estimate is allocated to the boroughs and census areas in proportion to the civilian population.

Disaster relief benefits.--These benefits are the Federal payments to the victims of disasters, such as hurricanes and riots.

The State and county estimates are based on information from the Federal Emergency Management Agency. The estimates for 1992 include payments to the victims of Hurricanes Andrew and Iniki; for 1993, the midwest floods; for 1994, the southern floods; and for 1994-96, the Northridge earthquake and floods in California.

Radiation exposure payments.--These are payments made under the Radiation Exposure Compensation Act, which offers compensation to individuals exposed to radiation released during above-ground nuclear weapons tests and uranium mining.

The State estimates are based on direct data from the Department of Justice. The State estimates are allocated to counties in proportion to the civilian population.

Japanese interns redress benefits.--These benefits, which began in 1990, are the payments to the American citizens of Japanese descent who were interned during World War II.

The State and county estimates are based on the tabulations of these payments by ZIP Code area from the Department of Justice. These tabulations are summed to counties by BEA.

Payment of anti-terrorism judgments.-- These are payments from the U.S. Treasury to satisfy certain court judgments against countries found to have sponsored terrorism.

In the absence of any pertinent data, the national estimates are allocated to States and counties in proportion to the civilian population.

Federal educational exchange benefits.--These benefits are payments to the students who participate in the Fulbright scholarship program and in other international educational exchange programs.

In the absence of any pertinent data, the national estimates are allocated to States and counties in proportion to the civilian population.

Bureau of Indian Affairs benefits.--These benefits are the payments to American Indians for educational and social services that are not available to them from State or local agencies.

The State estimates are based on data for these payments from the Bureau of Indian Affairs. The State estimates are allocated to counties in proportion to the American Indian population.

Government Payments to Nonprofit Institutions

These payments consist of the payments made by Federal, State, and local governments to nonprofit organizations that serve individuals. These payments exclude Federal Government payments for work under research and development contracts.

Federal Government payments

The national estimates of the Federal Government payments are allocated to States and counties in proportion to the civilian population.

State and local government payments

The State and local government payments consists of payments for education assistance and payments for employment and training.

The national estimates of the payments for education assistance are allocated to States by payments data published by the Census Bureau, and those for the payments for employment and training, by the civilian population. The State estimates of both categories of payments are allocated to counties in proportion to the civilian population.

Business Payments

Business transfer payments consist of payments to individuals and payments (corporate gifts) to nonprofit organizations that serve individuals.

Payments to individuals

These payments consist primarily of personal-injury liability payments to individuals other than employees.

Because no pertinent data are available, the national estimates are allocated to States and counties in proportion to the civilian population.

Payments to nonprofit organizations that serve individuals

The national estimates of the payments to nonprofit institutions are allocated to States in proportion to the estimates of the wages and salaries paid by membership organizations. The State estimates are allocated to counties in proportion to the civilian population.

Table F.--Relative Importance to Personal Income of Transfer Payments, by Component, United States, 2001

	Millions of dollars	Percent of personal income
Personal income..	8,677,490	100.00
Transfer payments..	1,171,083	13.50
Government payments to individuals..	1,111,824	12.81
Retirement & disability insurance benefit payments..	449,899	5.18
Old age, survivors, & disability insurance payments..	425,167	4.90
Railroad retirement and disability payments..	8,413	0.10
Workers' compensation benefits..	11,159	0.13
Other government disability insurance payments/1/..	5,160	0.06
Medical payments/2/..	475,838	5.48
Income maintenance benefit payments..	110,901	1.28
Supplemental security income (SSI) payments..	33,161	0.38
Family assistance/3/..	19,212	0.22
Food stamps..	16,006	0.18
Other income maintainance/4/..	42,522	0.49
Unemployment insurance benefit payments..	32,408	0.37
State unemployment insurance compensation..	31,532	0.36
Unemployment compensation of Federal civilian employees..	245	0.00
Unemployment compensation of railroad employees..	98	0.00
Unemployment compensation for veterans..	217	0.00
Other unemployment compensation/5/..	316	0.00
Veterans benefit payments..	26,467	0.31
Veterans pension and disability benefit payments..	23,261	0.27
Veterans readjustment payments/6/..	1,504	0.02
Veterans life insurance benefit payments..	1,692	0.02
Other assistance to veterans/7/..	10	0.00
Federal education and training assistance payments (other than for veterans)/8/..	13,141	0.15
Other payments to individuals/9/..	3,170	0.04
Government transfers to nonprofits..	25,881	0.30
Federal government payments..	8,461	0.10
State and local government payments/10/..	17,420	0.20
Business transfers..	33,378	0.38
Business transfer payments to individuals/11/..	23,855	0.27
Business transfer payments to nonprofit institutions..	9,523	0.11

1. Consists largely of temporary disability payments and black lung payments.

2. Consists of medicare, medicaid, and other medical vendor payments.

3. Consists of benefits--generally known as temporary assistance for needy families--provided under the Personal Responsibility and Work Opportunity Reconciliation Act of 1996.

4. Consists largely of general assistance, refugee assistance, foster home care

and adoption assistance, earned income tax credits, and energy assistance.

5. Consists of trade readjustment allowance payments, Redwood Park benefit payments, public service employment benefit payments, and transitional benefit payments.

6. Consists largely of veterans' readjustment benefit payments and educational assistance to spouses and children of disabled or deceased veterans.

7. Consists largely of payments to paraplegics, payments for autos and other conveyances for disabled veterans, veterans' aid, and veterans' bonuses.

8. Consists largely of Federal fellowship payments (National Science Foundation, fellowships and traineeships, subsistence payments to State maritime academy cadets, and other federal fellowships), interest subsidy on higher education loans, basic educational opportunity grants, and Job Corps payments.

9. Consists largely of Bureau of Indian Affairs payments; education exchange payments; Alaska Permanent Fund dividend payments; compensation of survivors of public safety officers; compensation of victims of crime; disaster relief payments; compensation for Japanese internment; and other special payments to individuals.

10. Consists of State and local government educational assistance payments to nonprofit institutions and other State and local government payments to nonprofit institutions.

11. Consists of personal injury payments to individuals other than employees and other business transfer payments.

NOTE.--Detail may not add to totals due to rounding.

Table G.--States for Which County-level Medicaid Payments Data are Available, 1991-2001

State	Year payments data most recently available
Arkansas	2001
Delaware	2000
Florida	2001
Georgia	2001
Hawaii	2001
Idaho	2001
Illinois	2001
Indiana	2001
Iowa	2001
Kansas	1991
Kentucky	2001
Louisiana	2000
Maryland	2001
Michigan	1999
Minnesota	2001
Mississippi	2001
Missouri	2001
Montana	2001
Nebraska	2001
Nevada	2001
New Hampshire	1997
New Jersey	2001
New Mexico	2001
New York	2001
North Carolina	2001
North Dakota	2001
Ohio	2001
Oklahoma	2001
Oregon	2001
Pennsylvania	2000
South Dakota	2001
Tennessee	2001
Texas	1996
Utah	2001
Virginia	2001
Washington	2001
Wisconsin	2001
Wyoming	2001
