# Supplementary material for: Victory Tax: A Holistic Income Tax System
Source: Entropy (Basel). 2021 Nov 11;23(11):1492. doi: 10.3390/e23111492 (PMC8624733; doi:10.3390/e23111492)
Supplement: Supplementary file 1 [file entropy-23-01492-s001.zip › bundle/results_to_give_away/supporting_gov_data/51361-HouseholdIncomeFedTaxes.pdf]

CONGRESS OF THE UNITED STATES
CONGRESSIONAL BUDGET OFFICE

CBO

The Distribution of Household Income and Federal Taxes, 2013

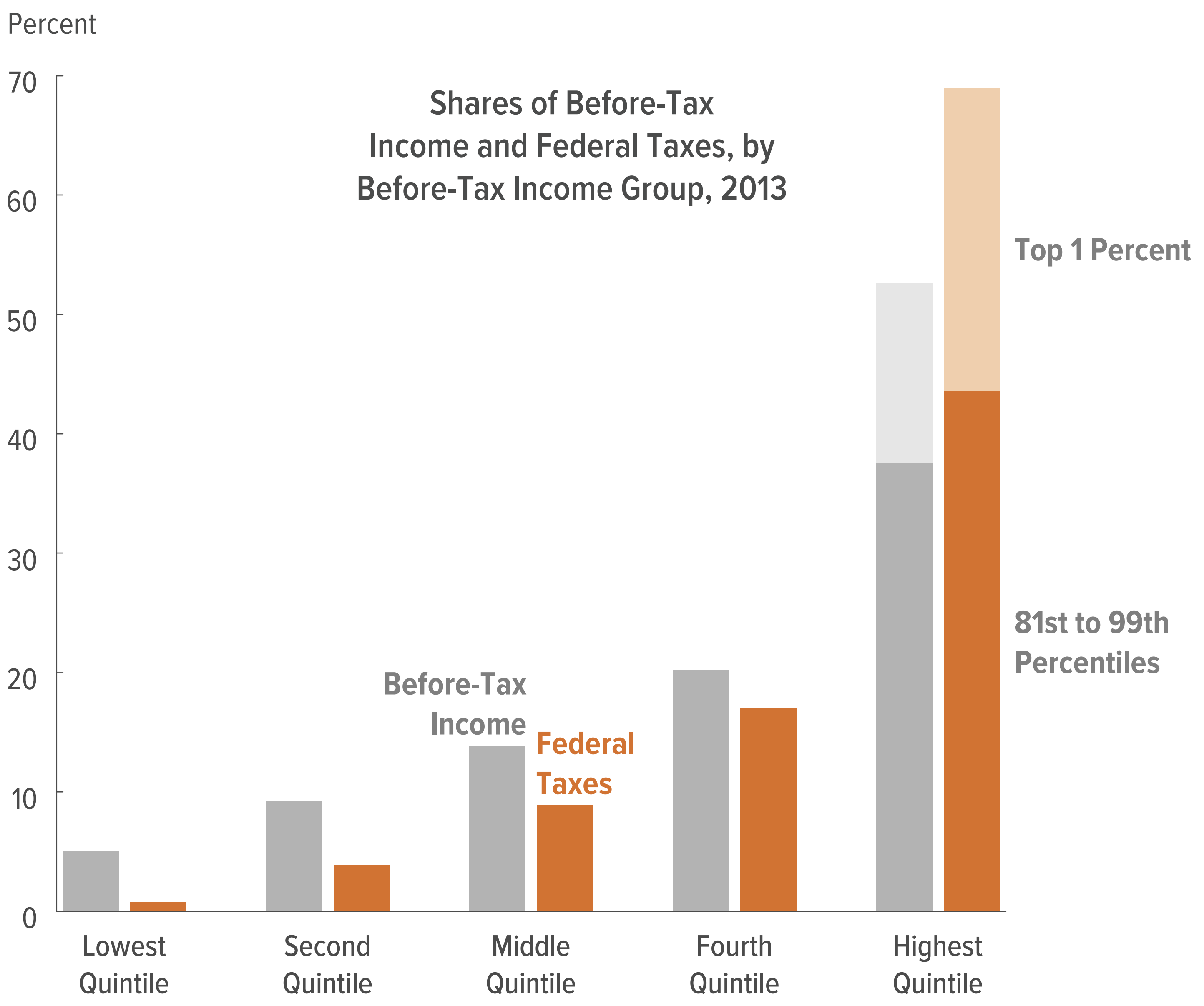

JUNE 2016

Notes and Definitions

Numbers in the text, tables, and figures may not add up to totals because of rounding. Dollar amounts are generally rounded to the nearest hundred.

Unless otherwise indicated, all years referred to in this report are calendar years.

More detailed notes and definitions related to this report are available in the appendix.

Market income consists of labor income, business income, capital gains (profits realized from the sale of assets), capital income excluding capital gains, income received in retirement for past services, and other sources of income.

Government transfers are cash payments and in-kind benefits from social insurance and other government assistance programs. Those transfers include payments and benefits from federal, state, and local governments.

Before-tax income is market income plus government transfers.

Federal taxes include individual income taxes, payroll (or social insurance) taxes, corporate income taxes, and excise taxes. In this analysis, those taxes for a given year are the amount a household owes on the basis of income received in that year, regardless of when the taxes are paid. Taxes from those four sources accounted for 93 percent of federal revenues in fiscal year 2013. Revenue sources not examined in this report include states' deposits for unemployment insurance, estate and gift taxes, customs duties, and miscellaneous receipts.

After-tax income is before-tax income minus federal taxes.

Average federal tax rates are calculated by dividing federal taxes by before-tax income.

Income groups are created by ranking households by their size-adjusted income. A household consists of people sharing a housing unit, regardless of their relationships. Each income quintile (fifth) contains approximately equal numbers of people but different numbers of households. Similarly, each percentile (hundredth) contains approximately equal numbers of people but different numbers of households. If a household has negative income (that is, if its business or investment losses are larger than its other income), it is excluded from the lowest income group but included in totals.

Household income over time is adjusted for inflation using the price index for personal consumption expenditures as calculated by the Bureau of Economic Analysis.

Some of the figures have shaded vertical bars that indicate the duration of recessions. (A recession extends from the peak of a business cycle to its trough.)

Supplemental data showing household income and average federal tax rates for households with children, elderly childless households, and nonelderly childless households are posted along with this report on CBO's website (www.cbo.gov/publication/51361).

Contents

Summary 1

How Were Income and Federal Taxes Distributed in 2013? 1

What Are the Trends in the Distribution of Income, Federal Taxes, and Income Inequality? 2

Income and Taxes Across Households in 2013 5

Market Income Across the Income Scale 6

Before-Tax Income Across the Income Scale 7

Changes to Federal Tax Rules in 2013 9

Federal Taxes Across the Income Scale 10

After-Tax Income Across the Income Scale 12

Trends in Household Income and Federal Taxes 14

Trends in Market Income 14

Trends in Before-Tax Income 17

Trends in Average Federal Tax Rates by Tax Source 18

Trends in Average Federal Tax Rates by Before-Tax Income Group 20

Trends in After-Tax Income 21

Trends in Income Inequality 21

Appendix: Data and Methods 25

List of Tables and Figures 29

About This Document 30

The Distribution of Household Income and Federal Taxes, 2013

Summary

In 2013, according to the Congressional Budget Office's estimates, average household market income—a comprehensive income measure that consists of labor income, business income, capital income (including capital gains), and retirement income—was approximately $86,000. Government transfers, which include benefits from programs such as Social Security, Medicare, and unemployment insurance, averaged approximately $14,000 per household. The sum of those two amounts, which equals before-tax income, was about $100,000, on average. In this report, CBO analyzed the distribution of four types of federal taxes: individual income taxes, payroll (or social insurance) taxes, corporate income taxes, and excise taxes. Taken together, those taxes amounted to about $20,000 per household, on average, in 2013.[1] Thus, average after-tax income—which equals market income plus government transfers minus federal taxes—was about $80,000, and the average federal tax rate (federal taxes divided by before-tax income) was about 20 percent.

How Were Income and Federal Taxes Distributed in 2013?

Before-tax income was unevenly distributed across households in 2013. Average before-tax income among households in the lowest one-fifth (or quintile) of the distribution of before-tax income was approximately $25,000 in 2013, CBO estimates (see Table 1). Among households in the middle income quintile, average before-tax income was about $70,000. Households in the highest income quintile had average before-tax income that was much higher—approximately $265,000. In response to tax law changes that went into effect in 2013, some taxpayers—especially those at the top of the income distribution—shifted some income into 2012 to avoid the higher tax rates on that income in 2013. Because of that income shifting, income at the top of the distribution was lower in 2013 than it would have been in the absence of those tax law changes.

Overall, federal taxes are progressive, meaning that average tax rates generally rise as income increases. Households in the lowest income quintile paid less than $1,000 in federal taxes in 2013, on average, which amounted to an average federal tax rate of about 3 percent, CBO estimates.[2] Households in the middle quintile paid about $9,000 in federal taxes, on average, and households in the highest quintile paid about $70,000; their average federal tax rates were approximately 13 percent and 26 percent, respectively.

Households in the highest income quintile received a little more than half of total before-tax income and paid more than two-thirds of all federal taxes in 2013 (see Figure 1). Households in other income groups received considerably smaller shares of before-tax income and paid considerably smaller shares of federal taxes. Furthermore, the share of taxes paid by households in other income groups (in contrast to households in the highest income quintile) was less than the share of before-tax income they received. Households in the middle income quintile, for instance, received about 14 percent of total before-tax income and paid about 9 percent of federal taxes. And households in the lowest income quintile received about 5 percent of before-tax income in 2013 and paid less than 1 percent of federal taxes, CBO estimates.

1. Data for 2013 are the most recent available with complete information about tax payments. For more details, see the appendix.

2. In this analysis, federal taxes are the amount a household owes based on income received in that year, regardless of when the taxes are paid. The term "paid" is used throughout the report for simplicity.

Table 1.

Average Household Income, Transfers, and Taxes, by Before-Tax Income Group, 2013

Dollars

	Lowest Quintile	Second Quintile	Middle Quintile	Fourth Quintile	Highest Quintile	All Households
Market Income	15,800	31,300	53,000	88,700	253,000	86,400
Government Transfers	9,600	16,200	16,700	15,000	12,000	13,900
Before-Tax Income	25,400	47,400	69,700	103,700	265,000	100,200
Federal Taxes	800	4,000	8,900	17,600	69,700	20,100
After-Tax Income	24,500	43,400	60,800	86,100	195,300	80,100

Source: Congressional Budget Office.

Market income consists of labor income, business income, capital gains (profits realized from the sale of assets), capital income excluding capital gains, income received in retirement for past services, and other sources of income.

Government transfers are cash payments and in-kind benefits from social insurance and other government assistance programs. Those transfers include payments and benefits from federal, state, and local governments.

Before-tax income is market income plus government transfers.

Federal taxes include individual income taxes, payroll taxes, corporate income taxes, and excise taxes.

After-tax income is before-tax income minus federal taxes.

Income groups are created by ranking households by before-tax income, adjusted for household size. Quintiles (fifths) contain equal numbers of people.

For more detailed definitions of income, see the appendix.

The progressive federal tax structure results in a distribution of after-tax income that is slightly more even than that of before-tax income. Households in the lowest income quintile received approximately 6 percent of after-tax income in 2013, compared with 5 percent of before-tax income, and households in the highest income quintile received about 49 percent of after-tax income, compared with 53 percent of before-tax income, CBO estimates.

What Are the Trends in the Distribution of Income, Federal Taxes, and Income Inequality?

The distributions of household income and federal taxes depend on economic conditions and tax laws—both of which have changed over time. Changes in the distribution of after-tax income can be traced to changes in the distributions of market income, government transfers, and federal taxes.[3]

Income inequality varies depending on the measure of income examined. By comparing measures of income inequality based on income with and without government transfers (market income and before-tax income), it is possible to determine the degree to which government transfers reduce income inequality. Similarly, by comparing measures of income inequality based on income excluding or including the effects of federal taxes (before-tax income and after-tax income), it is possible to determine the degree to which federal taxes reduce income inequality.

Market and Before-Tax Income. Over the 35-year period from 1979 to 2013, average inflation-adjusted market and before-tax income increased for each income group. The cumulative growth rates for those income measures, however, differed significantly across the income distribution. Market income in 2013 for households in the top 1 percent was 188 percent higher than it was in 1979. For households in the bottom four income quintiles, market income was 18 percent higher in 2013 than it was in 1979.

Because government transfers largely go to low-income households, the cumulative growth in before-tax income for households in the bottom four quintiles was significantly higher than the cumulative growth in market income for households in similar market income quintiles. In addition, because of such transfers, the average

3. Taxes and transfers can affect households' market income by creating incentives for people to change their behavior. If an additional dollar earned or saved leads to reductions in transfers or increases in taxes, then the after-tax return from working and saving is reduced, which may cause people to work or save less. However, those changes in transfers and taxes also reduce after-tax income, which may cause people to work or save more. In this analysis, CBO did not attempt to adjust market income to account for those behavioral effects.

Figure 1.

Shares of Before-Tax Income and Federal Taxes, by Before-Tax Income Group, 2013

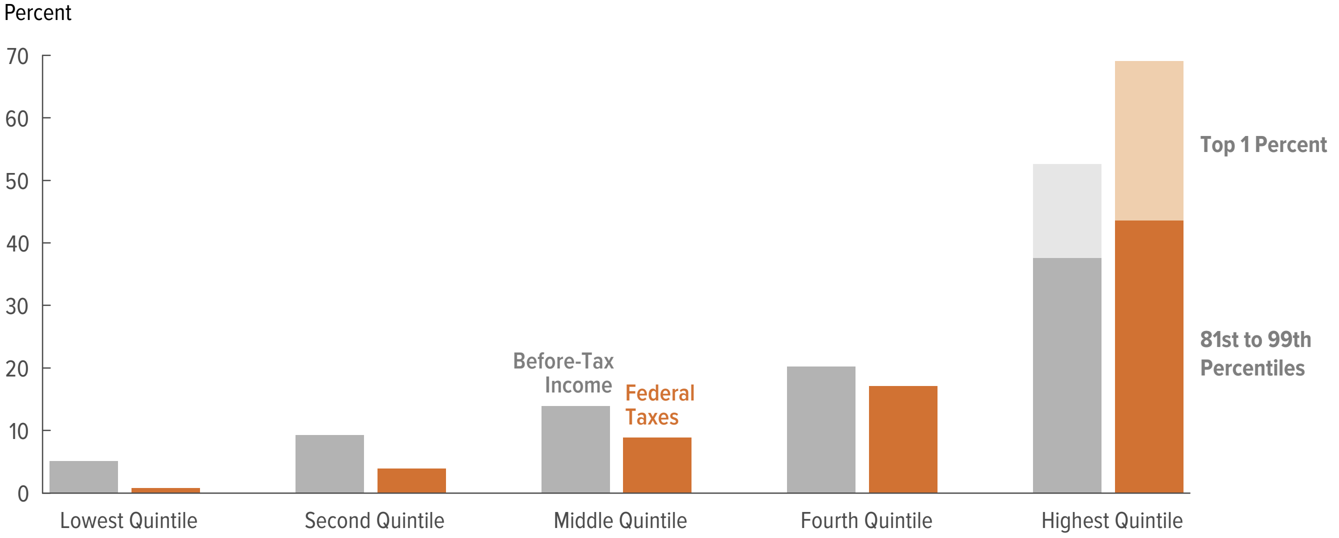

Source: Congressional Budget Office.

Before-tax income is market income plus government transfers. Market income consists of labor income, business income, capital gains (profits realized from the sale of assets), capital income excluding capital gains, income received in retirement for past services, and other sources of income. Government transfers are cash payments and in-kind benefits from social insurance and other government assistance programs. Those transfers include payments and benefits from federal, state, and local governments.

Federal taxes include individual income taxes, payroll taxes, corporate income taxes, and excise taxes.

Income groups are created by ranking households by before-tax income, adjusted for household size. Quintiles (fifths) contain equal numbers of people; percentiles (hundredths) contain equal numbers of people as well.

For more detailed definitions of income, see the appendix.

cumulative growth in before-tax income for households in the lowest quintile was slightly higher than the average cumulative growth for households in the middle three income quintiles—39 percent versus 32 percent. Conversely, because high-income households receive relatively small amounts of government transfers, on average, the inflation-adjusted cumulative growth rates over the 35-year period for market and before-tax income were very similar for those households.

Average Federal Tax Rates. Average federal tax rates fluctuate over time because of changes in tax law and changes in the composition and distribution of income. Primarily because of significant changes in tax rules, average federal tax rates in 2013 were higher than in 2012 across the income distribution. Households in the bottom 99 percent of the income distribution saw their average federal tax rates increase by more than 1 percentage point, on average, between 2012 and 2013. Households in the top 1 percent of the income distribution, however, saw their average federal tax rates jump by more than 5 percentage points between 2012 and 2013.[4] Those changes in average federal tax rates in 2013 made the federal tax system the most progressive it has been since at least the mid-1990s.

Despite the increases in tax rates in 2013, average federal tax rates in that year were below the 35-year average for most households. Average federal tax rates in 2013 for households in all but the top income quintile were significantly below the average rates over the 1979–2013 period. For the top quintile, the breakdown differs; the average tax rate in 2013 was slightly below the 35-year average for households in the 81st to 99th percentiles but well *above* the 35-year average for households in the top 1 percent of the income distribution (see Figure 2).

4. In addition to the distributional effects of those tax law changes, the overall increase in tax rates in 2013 had a significant impact on the federal revenues collected in fiscal year 2013. Between fiscal year 2012 and fiscal year 2013, total federal revenues increased by 13 percent. That increase—along with an 8 percent decrease in federal outlays—contributed to a reduction of more than $300 billion in the federal deficit between 2012 and 2013.

Figure 2.

Average Federal Tax Rates, by Before-Tax Income Group, 1979 to 2013

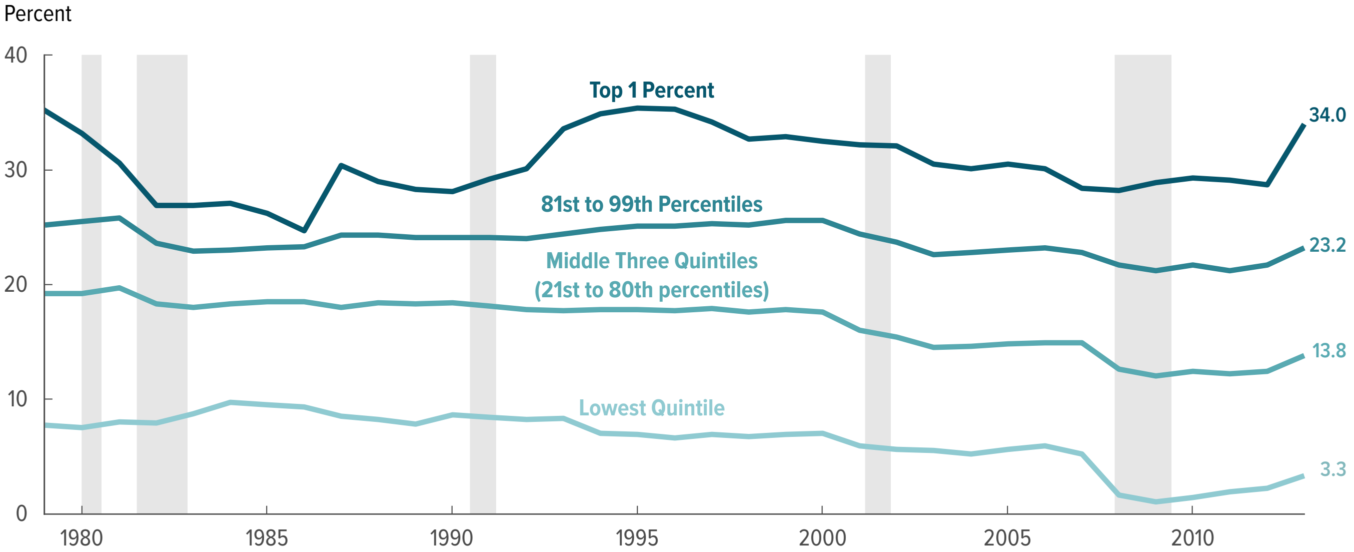

Source: Congressional Budget Office.

Average federal tax rates are calculated by dividing federal taxes by before-tax income.

Before-tax income is market income plus government transfers. Market income consists of labor income, business income, capital gains (profits realized from the sale of assets), capital income excluding capital gains, income received in retirement for past services, and other sources of income. Government transfers are cash payments and in-kind benefits from social insurance and other government assistance programs. Those transfers include payments and benefits from federal, state, and local governments.

Federal taxes include individual income taxes, payroll taxes, corporate income taxes, and excise taxes.

Income groups are created by ranking households by before-tax income, adjusted for household size. Quintiles (fifths) contain equal numbers of people; percentiles (hundredths) contain equal numbers of people as well

For more detailed definitions of income, see the appendix.

After-Tax Income. From 1979 to 2013, average after-tax income grew at significantly different rates for households at different points on the income scale.[5] For households in the top 1 percent of the income distribution, inflation-adjusted after-tax income grew at an average rate of about 3 percent per year, making that income 192 percent higher in 2013 than it was in 1979 for those households.[6] In contrast, households in the bottom quintile experienced an average growth of about 1 percent per year in their inflation-adjusted after-tax income over the same period, making that income 46 percent higher in 2013 than it was in 1979, CBO estimates. Those differences in growth rates for after-tax income are largely attributable to differences in growth rates for market income, although changes in taxes and transfers had an effect as well.

Income Inequality. Between 1979 and 2013, all three measures of income examined in this report—market income, before-tax income, and after-tax income—became less equally distributed, based on a standard measure of inequality known as the Gini index. The increase in inequality in both before-tax and after-tax income over the 35-year period stemmed largely from a significant increase in inequality in market income, mostly because of substantial income growth at the top of the market income distribution.

5. CBO chose 1979 as a starting point for the analysis because it is the earliest year for which the Census Bureau provides consistent estimates of some measures of income. The beginning and end points of the analysis—1979 and 2013—were different in terms of economic activity: 1979 was a peak year for the economy right before a recession, whereas 2013 was a year in which the economy had not yet fully recovered from the deep recession that ended in 2009.

6. The data used in this analysis contain income and tax information for a new set of households each year. Consequently, the households in a given income group in one year are not the same households in that same income group in the next year.

Because government transfers go predominantly to lower-income households, before-tax income (which is equal to market income plus government transfers) was more evenly distributed in each year than market income. And because higher-income households pay a larger share of federal taxes than lower-income households do, after-tax income was more evenly distributed than before-tax income.

In each year between 1979 and 2013, government transfers reduced income inequality significantly more than the federal tax system did. The effects of federal taxes have been relatively stable since the 1990s, whereas the effects of government transfers have generally fluctuated with the business cycle. The equalizing effects of government transfers increased significantly during the recession that began in 2007. Unlike previous economic cycles, government transfers have had a sustained effect on reducing income inequality during the subsequent slow recovery.

Income and Taxes Across Households in 2013

Throughout this report, CBO analyzes the distribution of three separate measures of income:

- Market income, which equals the sum of labor income, business income, capital income (including capital gains), and retirement income.[7]
- Before-tax income, which equals market income plus government transfers.[8]
- After-tax income, which equals before-tax income minus federal taxes.[9]

Average market income for households in 2013 was $86,400, CBO estimates.[10] On average, households received $13,900 in government transfers in that year—$9,900 from Social Security and Medicare and $4,000 from other government transfers. Before-tax income, on average, was therefore $100,200. In 2013, households paid an estimated $20,100 in federal taxes, resulting in average household after-tax income of $80,100. The average federal tax rate for that year (which is equal to federal tax liabilities divided by before-tax income) was 20.1 percent. That rate is the sum of four separate average tax rates: those for individual income taxes (9.2 percent), payroll taxes (7.7 percent), corporate income taxes (2.5 percent), and excise taxes (0.7 percent).

All of those measures—market income, government transfers, before-tax income, federal tax rates, and after-tax income—vary across the income scale, sometimes significantly. Market income is highly skewed toward the top of the income distribution: Households in the top quintile of the distribution of market income in 2013 received an estimated 59 percent of total market income, whereas households in the bottom quintile received an estimated 2 percent. Because government transfers constitute a larger share of market income toward the bottom of the income distribution, before-tax income is somewhat more evenly distributed across the income scale than market income—but it is still very skewed toward the top of the distribution. The progressivity of the federal tax system reduces income inequality further by disproportionately decreasing income at the top of the distribution. After-tax income, therefore, is more equally distributed across the income scale than before-tax income. Nonetheless, households in the top quintile of the distribution of before-tax income in 2013 received an estimated 49 percent of total after-tax income, whereas households in the bottom quintile received an estimated 6 percent.

7. See the appendix for more details on the sources of income included in market income.

8. Transfers as measured in this report do not equal total government spending on the transfer programs included in the analysis. The values of most transfers are based on amounts reported in the Census Bureau's Current Population Survey. The values of transfers from Medicare, Medicaid, and the Children's Health Insurance Program are based on the Census Bureau's estimate of the government's average cost of providing those benefits. In addition, because some transfers go to recipients outside the scope of the survey data collected by the Census Bureau and because some recipients misreport the amount of transfer payments they receive, the total amount of government transfers observed in the data used here is less than the total amount the government spends through those transfer programs. See the appendix for more details.

9. For this analysis, federal taxes include individual income taxes, payroll taxes, corporate income taxes, and excise taxes, which together accounted for 93 percent of all federal revenues in fiscal year 2013. Revenues from states' deposits for unemployment insurance, estate and gift taxes, miscellaneous fees and fines, and net income earned by the Federal Reserve, which make up the remaining 7 percent, are not allocated to households in this analysis, mainly because it is uncertain how to attribute those revenues to particular households.

10. Dollar amounts presented in this report are generally rounded to the nearest hundred.

Figure 3.

Average Market Income, by Market Income Group, 2013

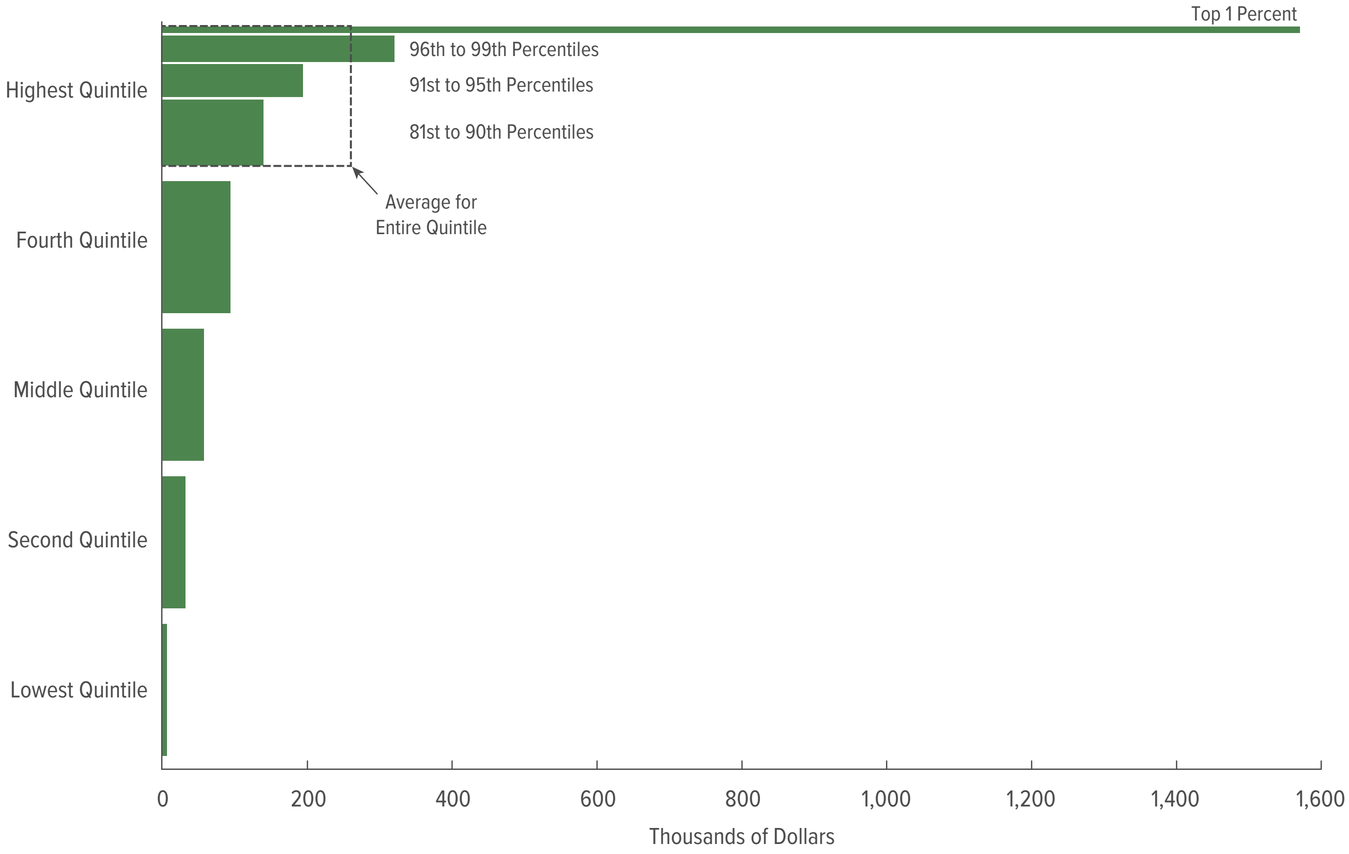

Source: Congressional Budget Office.

Market income consists of labor income, business income, capital gains (profits realized from the sale of assets), capital income excluding capital gains, income received in retirement for past services, and other sources of income.

Income groups are created by ranking households by market income, adjusted for household size. Quintiles (fifths) contain equal numbers of people; percentiles (hundredths) contain equal numbers of people as well.

For more detailed definitions of income, see the appendix.

Market Income Across the Income Scale

Market income is highly skewed toward households at the top of the income distribution (see Figure 3). In 2013, households in the lowest quintile of market income earned about $8,300 per household, on average.[11] Households in the middle quintile earned about $58,600, and households in the top quintile earned about $259,900. Within the top quintile of households, market income is also skewed toward the very top of the distribution. The 1.2 million households in the top 1 percent of the market income distribution earned about $1.6 million per household, on average, which accounted for approximately 17 percent of total market income in 2013.

The average income observed at the top of the income distribution in 2013, however, was affected by the tax law changes that significantly increased tax rates for that income group in that year. In anticipation of increased

11. Differences between the values presented in this section and those presented in Table 1 stem from differences in the income measure by which households are ranked. In Table 1, households are ranked by before-tax income, which includes income from government transfer programs, including Social Security and Medicare; in this section, households are ranked by market income, which excludes income from transfer programs. The different ranking means that the households in the lowest quintile of before-tax income are not the same as those in the lowest quintile of market income.

Table 2.

Components of Average Market Income, by Market Income Group, 2013

	Quintiles					All	Percentiles			Top 1
	Lowest	Second	Middle	Fourth	Highest	Households	81st–90th	91st–95th	96th–99th	Percent
Average Market Income (Dollars)	8,300	32,600	58,600	94,900	259,900	86,400	140,300	195,300	321,500	1,570,800
Share of Market Income (Percent)										
Labor income	66	75	80	82	65	73	82	80	70	36
Business income	11	5	3	3	12	8	4	6	12	23
Capital income and gains	5	4	3	3	16	11	5	6	12	38
Other income[a]	18	17	14	11	7	8	9	9	7	3

Source: Congressional Budget Office.

Income groups are created by ranking households by market income, adjusted for household size. Quintiles (fifths) contain equal numbers of people; percentiles (hundredths) contain equal numbers of people as well.

For more detailed definitions of income, see the appendix.

a. Includes income received in retirement for past services and other sources of income.

tax rates, high-income households probably shifted income from 2013 into 2012—especially income from sources for which there is flexibility in when the income is realized, such as capital gains. That shifting of income from 2013 into 2012 means that market income at the top of the income distribution was lower in 2013 than it would have been in the absence of those tax law changes.[12]

In 2013, labor income made up the largest share of market income for each income quintile (see Table 2). However, the share of market income derived from labor falls off significantly for households in the top 1 percent of the distribution (although the dollar amount continues to increase). Labor income accounts for an estimated 70 percent of market income among households in the 96th through 99th percentiles but only 36 percent of market income among households in the top 1 percent. Capital income (including realized capital gains) and business income increase significantly for the top 1 percent of households and therefore make up a much larger proportion of their market income, pushing down the share attributable to labor.

Before-Tax Income Across the Income Scale

Market income is by far the largest component of before-tax income for all income groups—ranging from an estimated 62 percent of before-tax income for households in the lowest quintile of before-tax income to about 99 percent of before-tax income for households in the top 1 percent (see Table 3).

Government transfers, the other component of before-tax income, include cash transfers (such as Social Security benefits and unemployment insurance benefits) and in-kind benefits (such as Medicare and Medicaid benefits).[13] Those transfers increase income across the entire distribution, on average, but make up a significantly larger share of before-tax income for lower-income groups than for

12. Preliminary data on the concentration of income at the top of the income distribution for 2014 indicate that, after the sharp increase in 2012 and sharp decrease in 2013, the share of income going to the top 1 percent of tax filers rebounded in 2014. That pattern is consistent with the shifting of income by taxpayers from 2013 into 2012 in anticipation of higher tax rates. Those data were originally reported in Thomas Piketty and Emmanuel Saez, "Income Inequality in the United States, 1913–1998," *Quarterly Journal of Economics*, vol. 118, no. 1 (February 2003), pp. 1–39, http://eml.berkeley.edu/~saez/pikettyqje.pdf (471 KB), but are regularly updated and available at http://eml.berkeley.edu/~saez/TabFig2014prel.xls (4 MB).

13. CBO recently undertook a more comprehensive analysis of the distribution of federal spending in 2006. Although that study used a similar methodology to the one used in this report, it differed in some important respects, most notably by adjusting the amount of transfer income reported in survey data to match the budgetary totals reported in the Treasury Department's *Monthly Treasury Statements*. The data used in this report are not aligned to budgetary totals and, because of underreporting of transfer income in surveys, do not capture the full effects of government transfers on household income. For more details, see Congressional Budget Office, *The Distribution of Federal Spending and Taxes in 2006* (November 2013), www.cbo.gov/publication/44698.

Table 3.

Components of Average Before-Tax Income, by Before-Tax Income Group, 2013

	Quintiles					All	Percentiles			Top 1
	Lowest	Second	Middle	Fourth	Highest	Households	81st–90th	91st–95th	96th–99th	Percent
Average Before-Tax Income (Dollars)	25,400	47,400	69,700	103,700	265,000	100,200	147,100	201,400	326,800	1,571,600
Share of Before-Tax Income (Percent)										
Market income	62	66	76	86	95	86	92	94	97	99
Labor income	53	57	63	69	60	62	73	72	66	35
Business income	6	3	2	2	11	7	3	5	11	23
Capital income and gains	1	1	2	3	16	10	5	7	12	38
Other income	2	5	9	11	8	7	11	10	8	3
Government transfers	38	34	24	14	5	14	8	6	3	1
Social Security	10	13	11	8	3	6	5	3	2	*
Medicare	9	9	7	4	1	4	2	2	1	*
Medicaid	10	8	4	1	*	2	*	*	*	*
Other cash and in-kind	9	5	3	2	*	2	1	1	*	*

Source: Congressional Budget Office.

Before-tax income is market income plus government transfers.

Market income consists of labor income, business income, capital gains (profits realized from the sale of assets), capital income excluding capital gains, income received in retirement for past services, and other sources of income.

Government transfers are cash payments and in-kind benefits from social insurance and other government assistance programs. Those transfers include payments and benefits from federal, state, and local governments.

Income groups are created by ranking households by before-tax income, adjusted for household size. Quintiles (fifths) contain equal numbers of people; percentiles (hundredths) contain equal numbers of people as well.

For more detailed definitions of income, see the appendix.

* = between 0 and 0.5 percent.

higher-income groups.[14] In 2013, government transfers accounted for more than one-third of before-tax income for households in the lowest and second-lowest income quintiles and about one-quarter of before-tax income for households in the middle quintile, CBO estimates. Those shares fall off significantly for higher-income households. Among households in the highest income quintile, about 5 percent of their before-tax income came from transfers; for households in the top 1 percent, that figure was less than 1 percent.

Social Security and Medicare are the two largest government transfer programs. Benefits from those programs are provided mostly to elderly households, many of which have low market income. Other transfers—including payments from the Supplemental Nutrition Assistance Program (SNAP) and benefits from Medicaid and the Children's Health Insurance Program—are provided even more disproportionately to households in the lower portion of the income distribution.

Although government transfers go predominantly to lower-income households, the fact that total transfers make up less than 15 percent of total before-tax income means that the distribution of before-tax income is only slightly less uneven than the distribution of market income. In 2013, according to CBO's estimates:

14. Transfers increase income most significantly for households at the bottom of the income distribution. Therefore, when households are ranked by before-tax income, which includes government transfers, households that are in the lowest market income quintile may be moved into higher before-tax income quintiles. Conversely, households in higher market income quintiles that receive less in transfers may move into lower before-tax income quintiles. Because of that reranking, income groups based on before-tax income are not directly comparable with income groups based on market income.

- Households in the lowest quintile received 5 percent of total before-tax income, or about $25,400 per household (see Figure 1 on page 3 and Table 1 on page 2).

- Households in the middle fifth received 14 percent of the total, or about $69,700 per household.

- Households in the top quintile received 53 percent of the total, or about $265,000 per household.

Before-tax income within the top quintile is also skewed toward the very top of the income distribution. Average before-tax income among households in the 81st through 90th percentiles (the bottom half of the top quintile) was $147,100, whereas before-tax income among households in the top 1 percent was about $1.6 million, on average, CBO estimates.

Changes to Federal Tax Rules in 2013

Average federal tax rates and after-tax income in 2013 were affected by several significant changes to federal tax rules that took effect in that year. Those changes affected not only the distribution of taxes but probably also the underlying distribution of income as households responded to the new tax rules. The three most significant changes in provisions of the individual income tax and payroll tax were the following:

- Several income tax provisions affecting high-income taxpayers, originally enacted in 2001 (in the Economic Growth and Tax Relief Reconciliation Act, or EGTRRA) and 2003 (in the Jobs and Growth Tax Relief Reconciliation Act, or JGTRRA), expired at the end of 2012;

- Payroll tax rates that had been reduced in 2011 and 2012 were reset to their previous, higher levels in 2013; and

- New taxes for high-income taxpayers enacted in the Affordable Care Act took effect in 2013.[15]

The American Taxpayer Relief Act of 2012, which was enacted in January 2013, made permanent most of the major provisions of EGTRRA and JGTRRA—with significant exceptions for high-income taxpayers. Specifically, for taxpayers earning in excess of $400,000 (for individual filers) or $450,000 (for married people filing jointly), the lower tax rates originally enacted in 2001 expired as scheduled, and the top statutory tax rate of 39.6 percent was reinstated. Also, the tax rate for long-term capital gains and dividends was increased from 15 percent to 20 percent for those high-income taxpayers, and limitations on the use of personal exemptions and itemized deductions for taxpayers whose adjusted gross income exceeded $250,000 (for individual filers) or $300,000 (for joint filers) were reinstated.

The Tax Relief, Unemployment Insurance Reauthorization, and Job Creation Act of 2010 reduced the employee's share of the Social Security payroll tax—from 6.2 percent to 4.2 percent—for 2011. That provision was extended through 2012 by two subsequent laws, but it was not extended into 2013. Consequently, in 2013, the reduction of 2 percentage points in the employee's share of the Social Security payroll tax expired, and the rate reverted to 6.2 percent.

Also taking effect in 2013 were two new taxes on high-income taxpayers that were implemented under the Affordable Care Act. For taxpayers earning more than $200,000 (for individual filers) or $250,000 (for joint filers), the Medicare payroll tax rate was increased by 0.9 percentage points. In addition, a 3.8 percent surtax was imposed on the lesser of a taxpayer's investment income (from interest, dividends, capital gains realizations, and certain passive business activity) and total income over certain thresholds ($200,000 for individual filers and $250,000 for joint filers).[16]

Several other changes in tax rules took effect in 2013 that probably had smaller effects on the distribution of income and taxes.[17] For example, under 2012 law, tax filers with qualifying investment properties could deduct 100 percent of their investment expenses. That full expensing provision (introduced in the Tax Relief, Unemployment

15. As used in this report, the Affordable Care Act comprises the Patient Protection and Affordable Care Act (Public Law 111-148), the health care provisions of the Health Care and Education Reconciliation Act of 2010 (P.L. 111-152), and the effects of subsequent judicial decisions, statutory changes, and administrative actions.

16. Additional tax and transfer provisions of the Affordable Care Act took effect in 2014, and some are scheduled to come into effect in later years.

17. The American Taxpayer Relief Act of 2012 also significantly modified estate and gift tax rules for 2013 relative to the rules specified for that year under prior law, increasing the exemption amount from $1 million to $5.25 million and reducing the top tax rate from 55 percent to 40 percent. Estate and gift taxes, however, are not included in this analysis.

Insurance Reauthorization, and Job Creation Act of 2010) expired at the end of 2012, and the depreciation schedule used for investments in 2013 was less generous than the full expensing in effect in 2011. Under the rules for 2013, depreciation deductions were smaller and taxable business income and taxes were higher than the corresponding amounts in 2012.

Federal Taxes Across the Income Scale

Average federal tax rates generally rise with income.[18] In 2013, households in the bottom fifth of the distribution of before-tax income paid an estimated 3.3 percent of their before-tax income in federal taxes, households in the middle quintile paid 12.8 percent, and households in the top quintile paid 26.3 percent. Average tax rates within the top quintile continued to increase as income rose: Households in the top 1 percent of the before-tax income distribution had an average tax rate of 34.0 percent (see Figure 4). As a result of the changes in tax rules that went into effect in 2013, average federal tax rates in that year were almost 2 percentage points higher than those in 2012.

Because the federal tax system overall is progressive, the share of taxes paid by higher-income households exceeds their share of before-tax income, and the opposite is true for lower-income households (see Figure 1 on page 3). In 2013, households in the highest quintile received an estimated 52.6 percent of before-tax income and paid 69.0 percent of federal taxes; households in the top 1 percent received 15.0 percent of before-tax income and paid 25.4 percent of federal taxes. In all other quintiles, the share of federal taxes was smaller than the share of before-tax income: Households in the bottom quintile received 5.1 percent of income and paid 0.8 percent of taxes, and households in the middle quintile received 13.9 percent of income and paid 8.9 percent of taxes.

Individual Income Taxes. Most of the progressivity of the federal tax system derives from the individual income tax. In 2013, households in the lowest quintile of before-tax income had an average tax rate for the individual income tax of -7.2 percent, and households in the second quintile had a rate of -1.2 percent, CBO estimates. (An income quintile has a negative average income tax rate if refundable tax credits in that quintile exceed other income tax liabilities.)[19] The average individual income tax rate was 2.6 percent for the middle quintile, 6.1 percent for the fourth quintile, and 15.5 percent for the highest quintile (see Figure 5). Households in the top 1 percent of the income distribution paid 23.6 percent of their before-tax income in individual income taxes, on average.[20]

Payroll Taxes. Average rates for payroll taxes are very similar across most of the income distribution but lower at the top of the distribution. In 2013, the average payroll tax rate was 8.0 percent for households in the lowest quintile of before-tax income, 7.6 percent for the second quintile, 8.3 percent for the middle quintile, and 9.0 percent for the fourth quintile, CBO estimates. The rate for the highest income quintile was 6.7 percent. The average rate among households in the top percentile was significantly lower—2.4 percent—than the rates for all other households, in part because more of the earnings for those households were above the maximum earnings subject to Social Security payroll taxes ($113,700 in 2013) and in part because earnings were a smaller share of their total income.

Among households in the top income quintile, the payroll tax rate is less than half the individual income tax rate, on average. For households in the bottom four quintiles, however, payroll tax rates are significantly higher than individual income tax rates. Payroll tax rates are almost 3 percentage points higher among households in the fourth quintile and almost 6 percentage points higher

18. Because of the complexity of estimating state and local taxes for individual households, this report considers federal taxes only. Researchers differ about whether state and local taxes are, on net, regressive, proportional, or slightly progressive, but most agree that state and local taxes are less progressive than federal taxes. For estimates of the distribution of state and local taxes, see Gerald Prante and Scott A. Hodge, *The Distribution of Tax and Spending Policies in the United States,* Special Report No. 211 (Tax Foundation, November 2013), http://tinyurl.com/k3lmxt5; and Carl Davis and others, *Who Pays? A Distributional Analysis of the Tax Systems in All 50 States*, 5th ed. (Institute on Taxation and Economic Policy, January 2015), www.itep.org/pdf/whopaysreport.pdf (1.4 MB).

19. In the federal budget, the refundable portion of individual income tax credits is treated as an outlay. In this analysis, CBO considers the refundable portion of the earned income tax credit and the child tax credit to be a negative tax liability rather than an outlay.

20. In addition to varying *across* income groups, average tax rates can vary significantly *within* income groups. The variation within income groups is attributable to several factors, including differences in the composition of income, family structure, and use of tax preferences, as well as the progressive rate structure. For more discussion of the variation in average tax rates within income groups, see Congressional Budget Office, *The Distribution of Household Income and Federal Taxes, 2010* (December 2013), www.cbo.gov/publication/44604.

Figure 4.

Average Federal Tax Rates, by Before-Tax Income Group, 2013

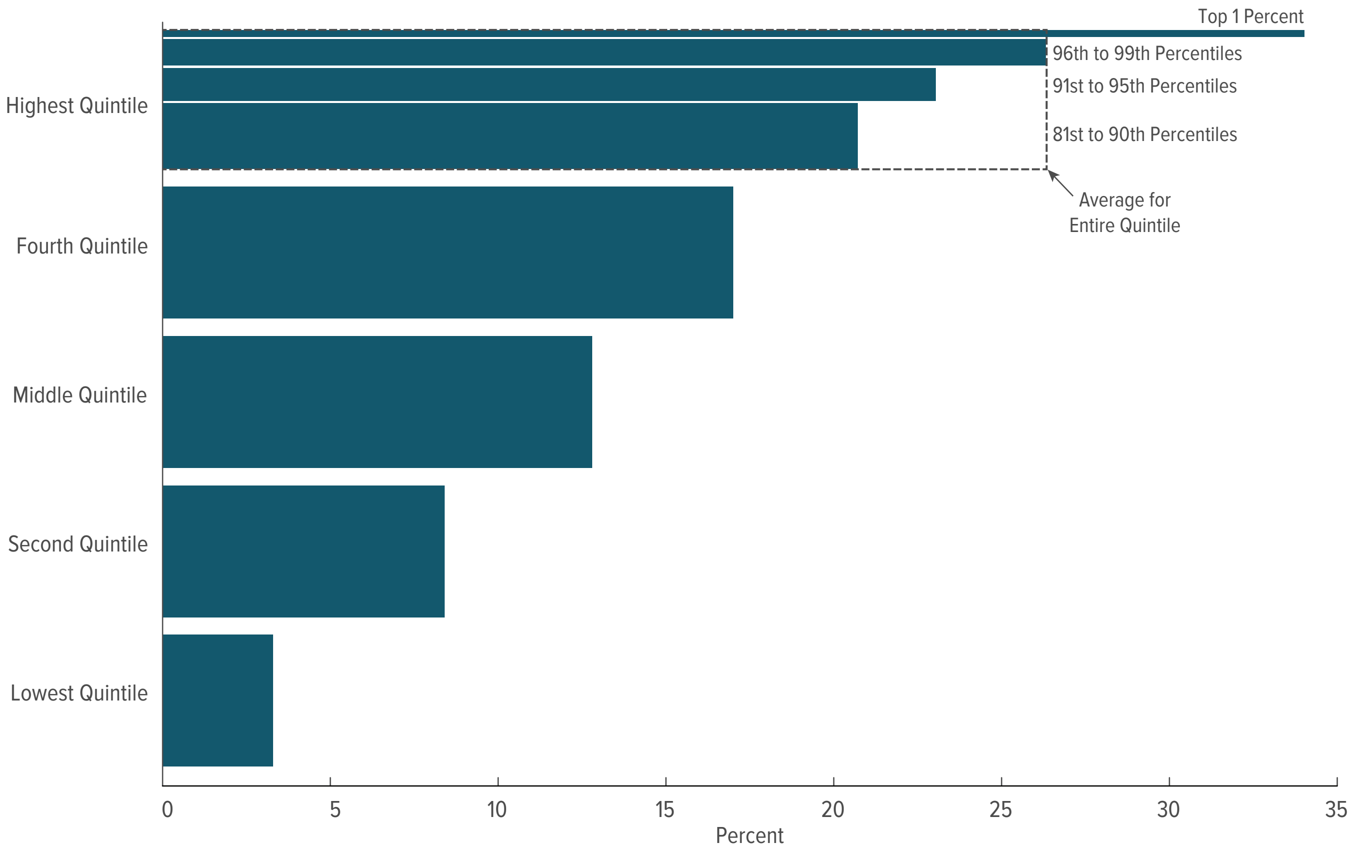

Source: Congressional Budget Office.

Average federal tax rates are calculated by dividing federal taxes by before-tax income.

Before-tax income is market income plus government transfers. Market income consists of labor income, business income, capital gains (profits realized from the sale of assets), capital income excluding capital gains, income received in retirement for past services, and other sources of income. Government transfers are cash payments and in-kind benefits from social insurance and other government assistance programs. Those transfers include payments and benefits from federal, state, and local governments.

Federal taxes include individual income taxes, payroll taxes, corporate income taxes, and excise taxes.

Income groups are created by ranking households by before-tax income, adjusted for household size. Quintiles (fifths) contain equal numbers of people; percentiles (hundredths) contain equal numbers of people as well.

For more detailed definitions of income, see the appendix.

for households in the middle quintile. Because individual income tax rates are negative, on average, for households in the bottom two quintiles, the differences between payroll tax rates and income tax rates are even more significant. Payroll tax rates are about 9 percentage points and 15 percentage points higher than income tax rates for households in the second and lowest quintiles, respectively.

Corporate Income Taxes. The average corporate income tax borne by households increases with income. CBO allocates most of that tax in proportion to each household's share of total capital income (including adjusted capital gains), which constitutes a larger share of income at the top of the distribution.[21] In 2013, the average corporate income tax rate—corporate taxes divided by before-tax household income—was 3.7 percent for households in the highest quintile and around

21. CBO allocates 75 percent of the corporate income tax to households in proportion to their share of capital income and 25 percent to households in proportion to their share of labor income. For more discussion of the incidence of the corporate income tax, see Congressional Budget Office, *The Distribution of Household Income and Federal Taxes, 2008 and 2009* (July 2012), www.cbo.gov/publication/43373. For more discussion of the adjustments made to realized capital gains when allocating the corporate tax to households, see the appendix.

Figure 5.

Average Federal Tax Rates, by Before-Tax Income Group and Tax Source, 2013

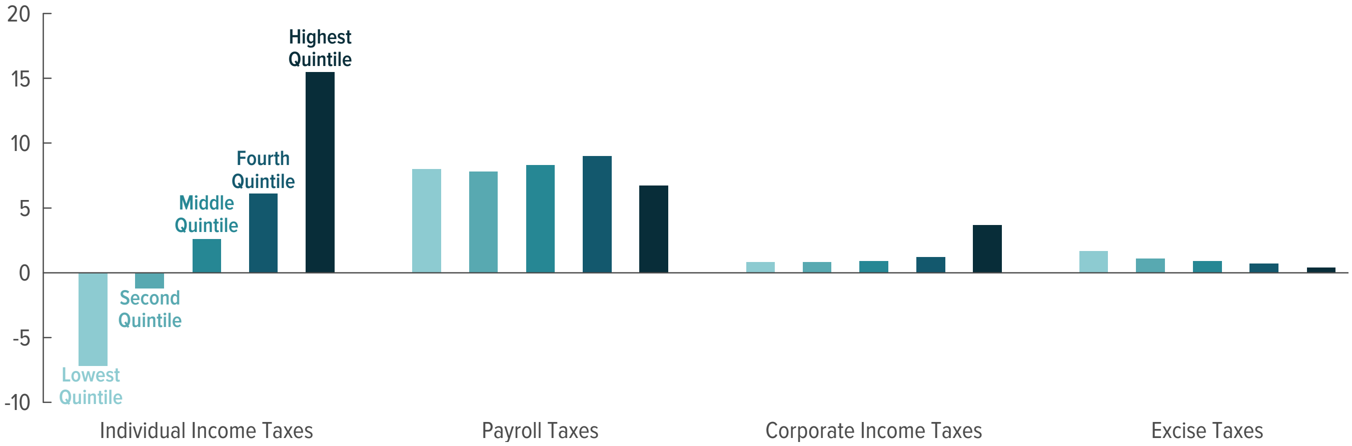

Source: Congressional Budget Office.

Average federal tax rates are calculated by dividing federal taxes by before-tax income.

Before-tax income is market income plus government transfers. Market income consists of labor income, business income, capital gains (profits realized from the sale of assets), capital income excluding capital gains, income received in retirement for past services, and other sources of income. Government transfers are cash payments and in-kind benefits from social insurance and other government assistance programs. Those transfers include payments and benefits from federal, state, and local governments.

Negative average tax rates for individual income taxes result when refundable tax credits, such as the earned income tax credit and the child tax credit, exceed the other income tax liabilities of the households in an income group.

Income groups are created by ranking households by before-tax income, adjusted for household size. Quintiles (fifths) contain equal numbers of people; percentiles (hundredths) contain equal numbers of people as well.

For more detailed definitions of income, see the appendix.

1 percent for households in the other four income quintiles, CBO estimates. In that year, almost 80 percent of the total corporate tax burden was borne by households in the highest income quintile; about 47 percent of the total corporate tax burden was borne by households in the top 1 percent of the income distribution.

Excise Taxes. Sales of a wide variety of goods and services are subject to federal excise taxes. Most of the revenues raised come from taxes on the sale of motor fuels (gasoline and diesel fuel), tobacco products, alcoholic beverages, and aviation-related goods and services (such as aviation fuel and airline tickets). Excise taxes are regressive—that is, the burden of excise taxes relative to income is greatest for lower-income households, which tend to spend a larger share of their income on those taxed goods and services. In 2013, average excise tax rates were 1.7 percent for households in the lowest income quintile, 0.9 percent for households in the middle income quintile, and 0.4 percent for households in the highest income quintile, CBO estimates.

After-Tax Income Across the Income Scale

In 2013, households in each income group paid a positive amount of federal taxes, on average. Consequently, average after-tax income was lower than average before-tax income for each income group. Because average federal tax rates rise with income, the difference between before-tax and after-tax income grows as income rises, and the distribution of after-tax income is slightly more even than the distribution of before-tax income. In the lowest quintile of before-tax income, average after-tax income was more than $800 lower than average before-tax income ($24,500 versus $25,400); for households in the middle quintile of before-tax income, the difference was approximately $8,900 ($60,800 versus $69,700); for households in the highest quintile of before-tax income, the difference was approximately $69,700 ($195,300 versus $265,000); see Table 1 on page 2. For households in the top 1 percent, the difference was approximately $534,000 ($1,037,000 versus $1,572,000), CBO estimates.

Another metric used to examine how the distributions of before- and after-tax income differ is the differences in the shares of those income measures going to each

Figure 6.

Shares of Before-Tax and After-Tax Income, by Before-Tax Income Group, 2013

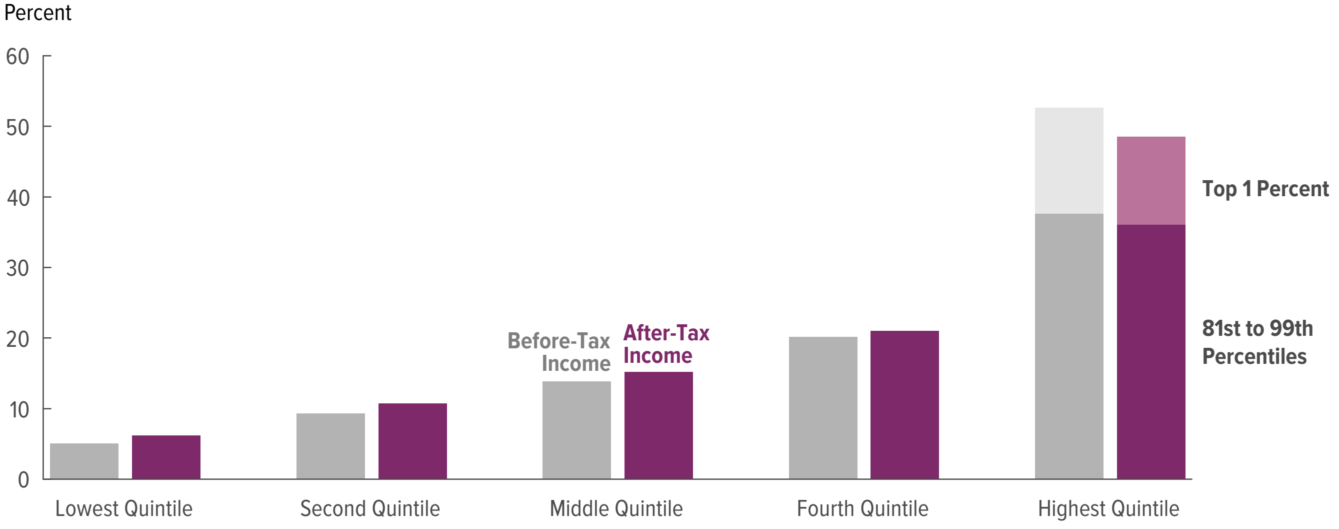

Source: Congressional Budget Office.

Before-tax income is market income plus government transfers. Market income consists of labor income, business income, capital gains (profits realized from the sale of assets), capital income (excluding capital gains), income received in retirement for past services, and other sources of income. Government transfers are cash payments and in-kind benefits from social insurance and other government assistance programs. Those transfers include payments and benefits from federal, state, and local governments.

After-tax income is before-tax income minus federal taxes. Federal taxes include individual income taxes, payroll taxes, corporate income taxes, and excise taxes.

Income groups are created by ranking households by before-tax income, adjusted for household size. Quintiles (fifths) contain equal numbers of people; percentiles (hundredths) contain equal numbers of people as well.

For more detailed definitions of income, see the appendix.

income group (see Figure 6). According to CBO's estimates, households in the bottom four quintiles of the distribution of before-tax income received shares of after-tax income that were roughly 1 percentage point larger than their shares of before-tax income. In contrast, for households in the top quintile, the share of after-tax income was 4 percentage points *lower* than the share of before-tax income. Most of that difference for the top income quintile was attributable to the difference between the shares of before-tax and after-tax income for the top 1 percent of the income distribution. Households in that income group received an estimated 15 percent of before-tax income and 12 percent of after-tax income.

After-tax income also reflects the impact of government transfers on the distribution of income. The distributional effects of the combination of government transfers and federal taxes can be seen by examining differences in the distributions of market income (income before government transfers and federal taxes) and after-tax income (see Figure 7).[22] Those differences were most significant for households in the lowest and highest quintiles of market income. Households in the lowest income quintile had a share of after-tax income that was 7 percentage points higher than their share of market income—9 percent versus 2 percent. In contrast, households in the highest income quintile had a share of after-tax income that was 11 percentage points *lower* than their share of market income—48 percent versus 59 percent. The differences in the shares of market income and after-tax income were not as large for households in the other three quintiles. Households in the second and middle quintiles had larger shares of after-tax income than market income, whereas households in the fourth quintile had a smaller share of after-tax income than market income.

22. Differences in the distributions of after-tax income shown in Figure 6 and Figure 7 are attributable to differences in the income measure used to rank households in the two figures. Households are ranked by before-tax income adjusted for household size in Figure 6 and by market income adjusted for household size in Figure 7.

Figure 7.

Shares of Market and After-Tax Income, by Market Income Group, 2013

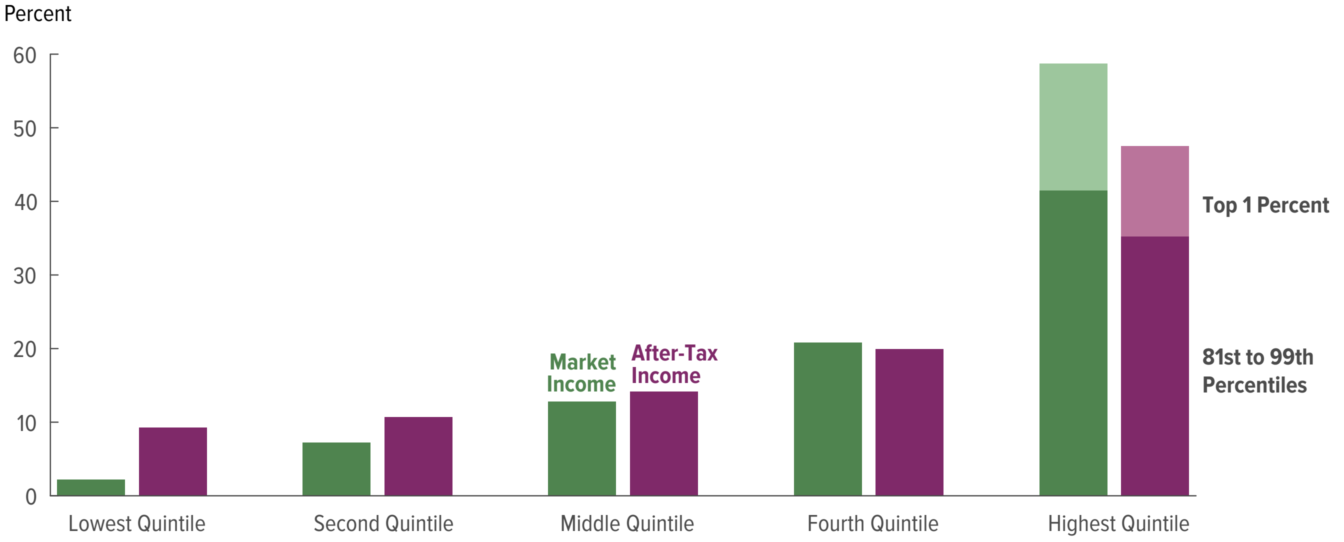

Source: Congressional Budget Office.

Market income consists of labor income, business income, capital gains (profits realized from the sale of assets), capital income excluding capital gains, income received in retirement for past services, and other sources of income.

After-tax income is market income plus government transfers minus federal taxes. Government transfers are cash payments and in-kind benefits from social insurance and other government assistance programs. Those transfers include payments and benefits from federal, state, and local governments. Federal taxes include individual income taxes, payroll taxes, corporate income taxes, and excise taxes.

Income groups are created by ranking households by market income, adjusted for household size. Quintiles (fifths) contain equal numbers of people; percentiles (hundredths) contain equal numbers of people as well.

For more detailed definitions of income, see the appendix.

Trends in Household Income and Federal Taxes

Over time, shifting economic conditions as well as changes in government transfers and federal taxes have led to changes in the distribution for each of the three income measures examined in this report—market income, before-tax income, and after-tax income. The amount of federal taxes paid by households each year, and thus their after-tax income, depends on shifts in the composition and distribution of income, changes in tax law, and households' responses to those tax law changes.

Trends in Market Income

The growth in market income from 1979 to 2013 varied significantly across the income scale. Over the entire 35-year period, cumulative growth in inflation-adjusted market income was 18 percent for households in the bottom four quintiles but much greater for households in the top quintile, CBO estimates (see Figure 8). Specifically, cumulative growth in inflation-adjusted market income for households in the 81st through 99th percentiles was 63 percent over the period, and cumulative growth for households in the top 1 percent of the distribution was 188 percent over the period. Average market incomes in 2013 were still below their peaks in 2007—before the recent deep recession—for each income group.

Fluctuations in the growth of market income result partly from the ups and downs of the business cycle and partly from the responses of taxpayers to changes in tax law. For households in the top 1 percent of the distribution, the peaks in market income in 2000 and 2007 preceded sharp drops in capital income as the economy entered recessions. The sharp rise and subsequent drop in market income in 2012 and 2013, however, were most likely caused by the shifting of income by taxpayers out of 2013 and into 2012 to avoid the higher tax rates that went into effect in 2013.[23]

23. A similar pattern occurred in 1986 and 1987, when taxpayers were also responding to significant changes in tax law (see Figure 8). Although there is evidence of income shifting in response to other tax law changes, those responses were smaller and are not as evident in Figure 8.

Figure 8.

Cumulative Growth in Average Inflation-Adjusted Market Income, by Market Income Group, 1979 to 2013

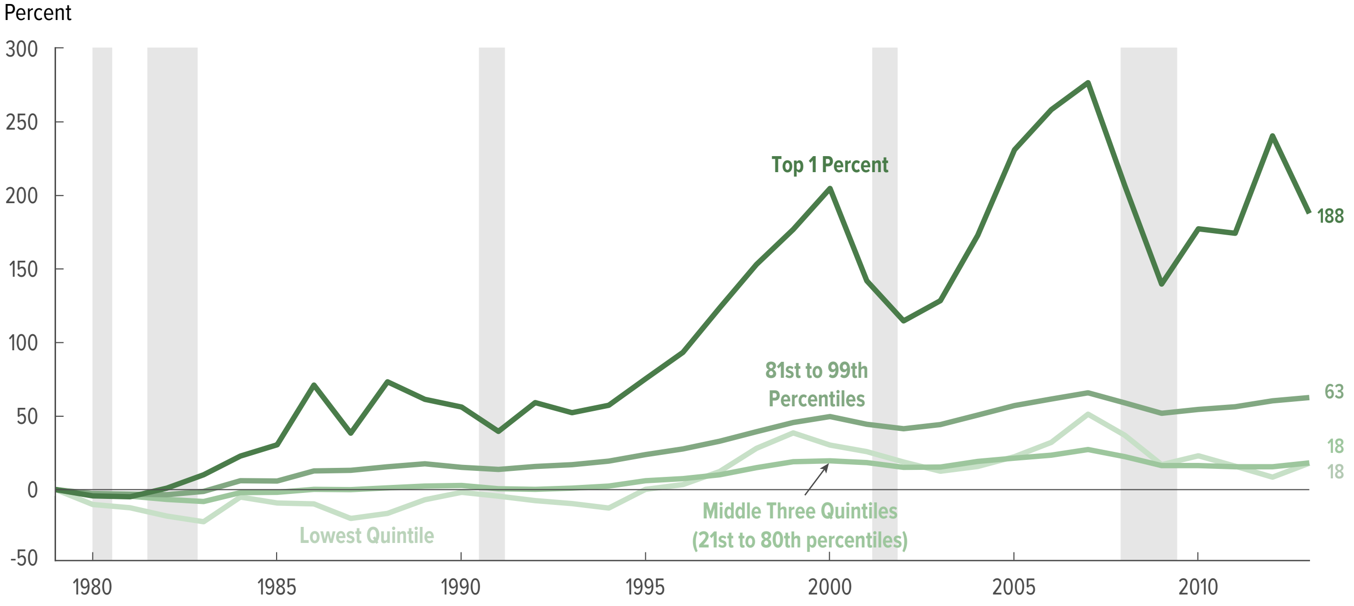

Source: Congressional Budget Office.

Market income consists of labor income, business income, capital gains (profits realized from the sale of assets), capital income excluding capital gains, income received in retirement for past services, and other sources of income. Before-tax income is market income plus government transfers. Government transfers are cash payments and in-kind benefits from social insurance and other government assistance programs. Those transfers include payments and benefits from federal, state, and local governments.

Income is converted to 2013 dollars using the price index for personal consumption expenditures.

Income groups are created by ranking households by market income, adjusted for household size. Quintiles (fifths) contain equal numbers of people; percentiles (hundredths) contain equal numbers of people as well.

For more detailed definitions of income, see the appendix.

Income from capital gains is especially sensitive to changes in law that affect tax rates on capital gains, because taxpayers have considerable flexibility in determining when they will sell their assets and realize taxable gains.[24] For households in the top 1 percent of the distribution of market income, for example, capital gains increased by about 67 percent between 2011 and 2012 and fell by 34 percent between 2012 and 2013. Such sizable shifts are unlikely to represent permanent changes in income from capital gains among households in the top 1 percent. Households in that portion of the income distribution, however, may have reduced their income from capital gains—and other components of market income—in more permanent ways in response to the higher tax rates that took effect in 2013.

For households not in the top 1 percent of the income distribution, the composition of market income by source was relatively stable between 1979 and 2013. However, the composition of market income changed markedly over time for households in the top 1 percent (see Figure 9). Their labor income and business income grew especially rapidly. Average labor income among households in the top 1 percent of the distribution of market income in the early 1980s was equal to about $200,000, adjusted for inflation to 2013 dollars. Since 2010, that income has averaged more than $600,000 per year, CBO estimates. The growth of business income over the period has been even greater. That income grew from about $50,000 in the early 1980s to over $350,000, on average, between 2005 and 2013. Realized capital gains for households in the top 1 percent of the income distribution have been the most volatile source of income and have occasionally been the largest source of income for

24. In addition to realizing capital gains in advance of tax increases, income can be shifted in other ways in response to tax changes. For example, when changes in tax laws alter the relationship in relative marginal rates between the individual and corporate income tax systems, taxpayers may be motivated to shift income from one tax system to the other, depending on which has become more (or less) advantageous as a result of the tax law change.

Figure 9.

Components of Inflation-Adjusted Market Income for the Top 1 Percent of Households, 1979 to 2013

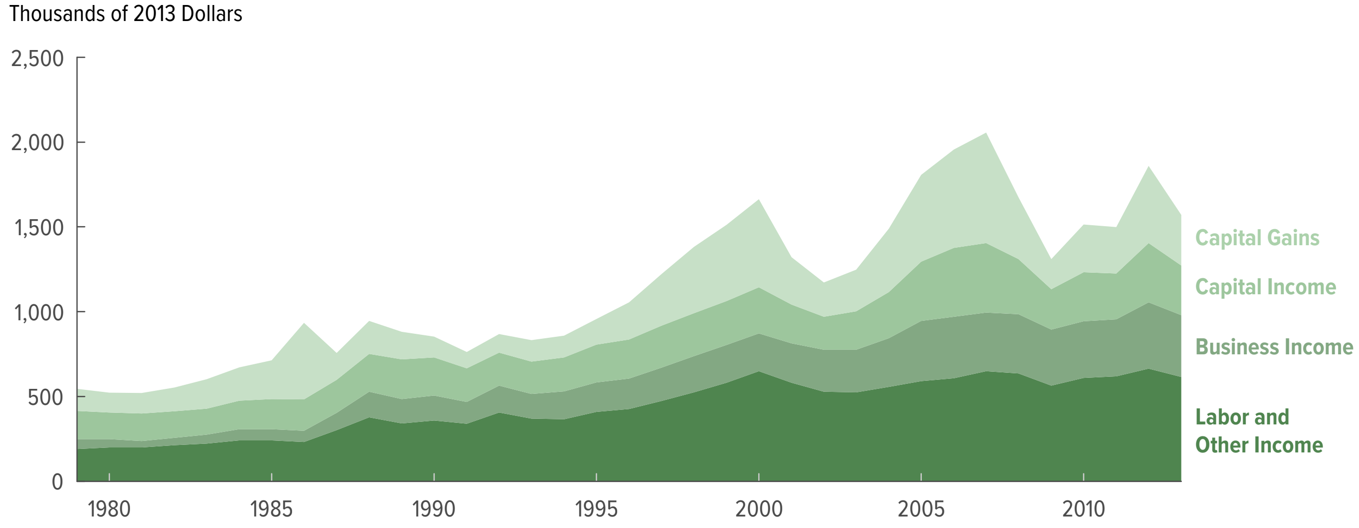

Source: Congressional Budget Office.

Other income includes income received in retirement for past services and other sources of income.

Income is converted to 2013 dollars using the price index for personal consumption expenditures.

Income groups are created by ranking households by market income, adjusted for household size.

For more detailed definitions of income, see the appendix.

this group. Much of the volatility in that income source is attributable either to behavioral responses to changes in tax laws (in 1986 and 2012, for example) or to significant expansions and contractions of asset prices (in 2001 and 2007, for example).

Because of the volatile nature of capital gains realizations and their significance in the income of the highest-income households, shifts in the relative importance of other sources of income for that group can be seen more clearly by examining market income excluding capital gains. From 1979 through the early 2000s, the share of that income for the top 1 percent of households that came from capital income excluding capital gains fell by half—from about 40 percent to about 20 percent, CBO estimates (see Figure 10). The share of that income taking the form of capital income turned back up in the early 2000s and then dropped again in the late 2000s as capital income fell during the last recession. In contrast, the share of market income excluding capital gains for the top 1 percent of households that came from business income has grown steadily, rising from 14 percent in 1979 to about 29 percent in 2013.

That shift in the composition of market income for households in the top 1 percent of the income distribution probably reflects, at least in part, significant changes in the organizational structure of businesses that have occurred over the past few decades. Following the Tax Reform Act of 1986, which lowered the top statutory tax rate on individual income below the top statutory tax rate on corporate income, many C corporations (which are taxed under the corporate income tax) were converted to S corporations (which pass corporate income through to their shareholders, who then pay individual income taxes on that income) or other types of entities not subject to the corporate income tax.[25] As a result, some income previously reported as capital gains and dividends (from C corporations) was instead reported as business income (from S corporations or other pass-through entities). That conversion also accelerated the realization of income, because profits of S corporations are required to be fully distributed to shareholders every year, whereas C corporations can retain their earnings. Business income jumped and capital income dropped over the 1986–1988 period as those conversions began, and the share of business income has continued to grow since then as more conversions occur and as new businesses are less likely to be set up as C corporations.

25. For more details on that shift, see *Taxing Businesses Through the Individual Income Tax* (December 2012), www.cbo.gov/publication/43750.

Figure 10.

Shares of Market Income Excluding Capital Gains, by Source, for the Top 1 Percent of Households, 1979 to 2013

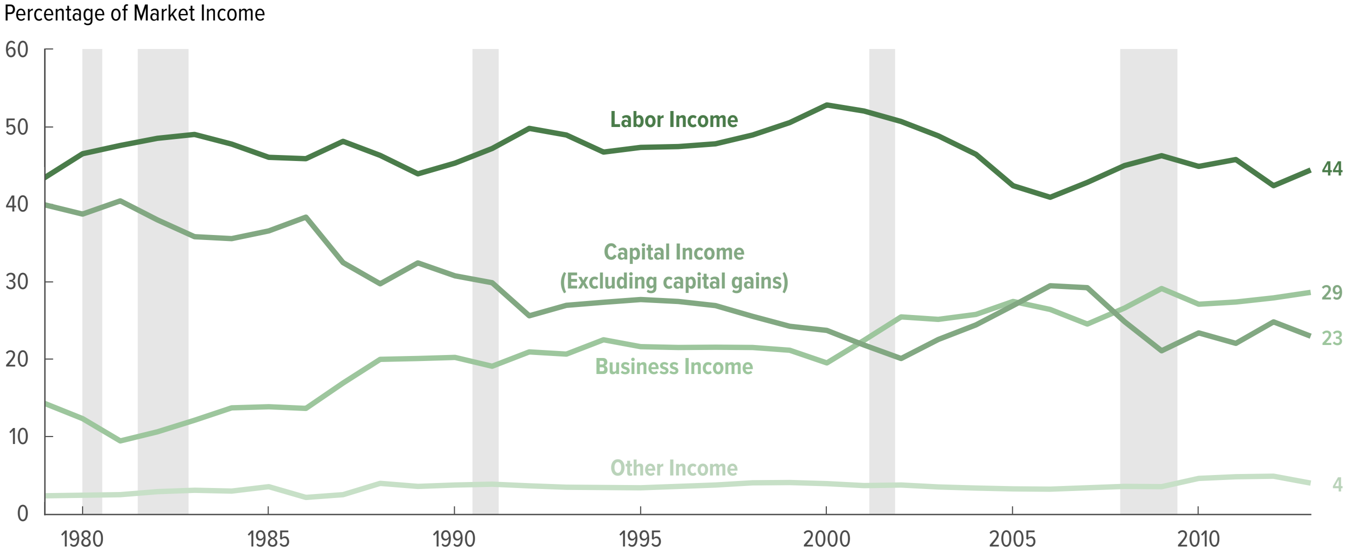

Source: Congressional Budget Office.

Other income includes income received in retirement for past services and other sources of income.

Income groups are created by ranking households by market income, adjusted for household size.

For more detailed definitions of income, see the appendix.

Trends in Before-Tax Income

Together, market income and government transfers constitute before-tax income. Government transfers, which include cash transfers (such as Social Security benefits and unemployment insurance benefits) and in-kind benefits (such as Medicare and Medicaid benefits), have increased significantly during the past few decades.[26] Those transfers accrue disproportionately to low-income households, so the cumulative growth in before-tax income exceeds the cumulative growth in market income by the largest amount for income groups toward the bottom of the income distribution.[27]

Between 1979 and 2013, the cumulative growth in inflation-adjusted before-tax income for households in the lowest quintile of before-tax income was 39 percent, CBO estimates (see Figure 11). For comparison, the cumulative growth in market income for households in the lowest quintile of market income was 18 percent over the same period (see Figure 8 on page 15).[28] Moreover, because of the effects of government transfers, the year-to-year volatility in income growth for households in the lowest before-tax income quintile was less than that for households in the lowest market income quintile.

For households in the middle three quintiles of before-tax income, the cumulative growth in inflation-adjusted before-tax income between 1979 and 2013 was 32 percent, which is also substantially higher than the market income growth for the similar market income group (18 percent). For households in the 81st through 99th percentiles and in the top 1 percent, inflation-adjusted before-tax income increased by an estimated 65 percent and 187 percent, respectively, from 1979 to 2013. Those cumulative growth rates are similar to the rates observed for market income

26. The government transfers included in before-tax income are mostly federal transfers, but they also include transfers from state and local governments—the largest of which is states' share of the cost of Medicaid benefits.

27. For an examination of trends in federal spending for means-tested transfer programs, see Congressional Budget Office, *Growth in Means-Tested Programs and Tax Credits for Low-Income Households* (February 2013), www.cbo.gov/publication/43934.

28. Because households receive different amounts of government transfers, the ranking of households based on market income differs from the ranking of households based on before-tax income. As a result, income groups that are based on ranking households by before-tax income include different households than the corresponding groups that are based on ranking households by market income.

Figure 11.

Cumulative Growth in Average Inflation-Adjusted Before-Tax Income, by Before-Tax Income Group, 1979 to 2013

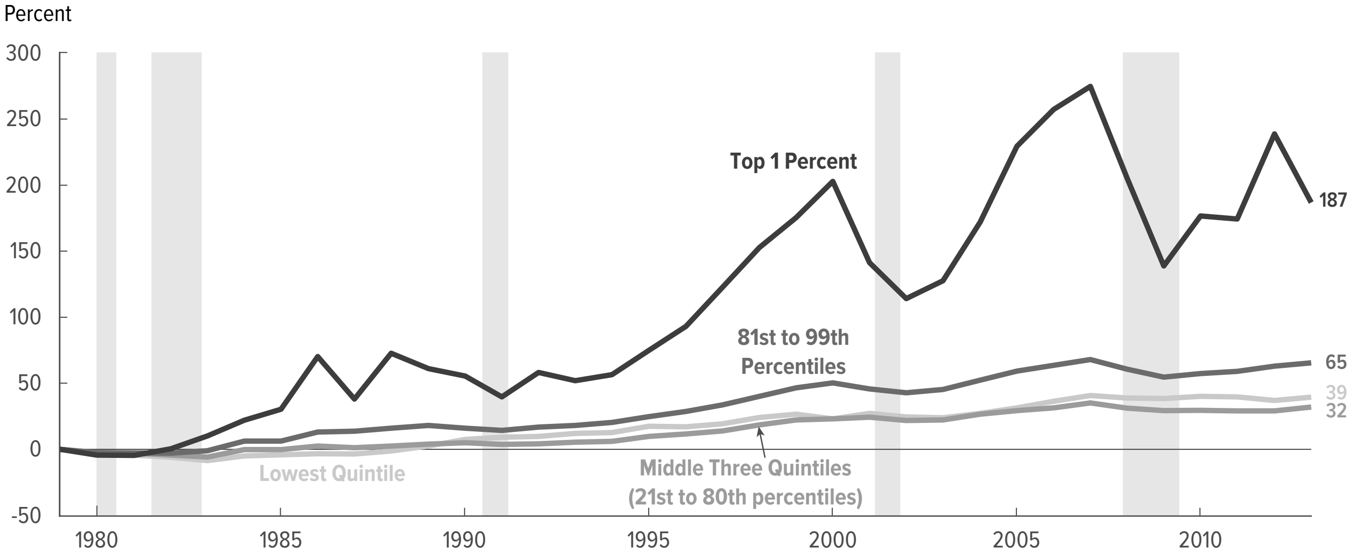

Source: Congressional Budget Office.

Before-tax income is market income plus government transfers. Market income consists of labor income, business income, capital gains (profits realized from the sale of assets), capital income excluding capital gains, income received in retirement for past services, and other sources of income. Government transfers are cash payments and in-kind benefits from social insurance and other government assistance programs. Those transfers include payments and benefits from federal, state, and local governments.

Income is converted to 2013 dollars using the price index for personal consumption expenditures.

Income groups are created by ranking households by before-tax income, adjusted for household size. Quintiles (fifths) contain equal numbers of people; percentiles (hundredths) contain equal numbers of people as well.

For more detailed definitions of income, see the appendix.

among households in the corresponding percentiles of the distribution of market income.

Trends in Average Federal Tax Rates by Tax Source

In 2013, households paid, on average, 20.1 percent of their before-tax income in federal taxes, CBO estimates (see Figure 12). That federal tax rate—which is equal to the sum of the rates for the individual income tax, payroll taxes, the corporate income tax, and excise taxes—is just below the average rate for the 1979–2013 period (20.8 percent). After a drop of roughly 2 percentage points in the early 1980s, primarily because of the combined effects of tax law changes and two recessions, the overall average tax rate rose steadily from the mid-1980s through the 1990s, reaching a peak in 2000 of 22.7 percent. That rate fell again in the early 2000s, largely because of tax legislation enacted in 2001 and 2003. The overall average tax rate climbed in the mid-2000s as economic conditions improved but then dropped between 2007 and 2008. The large decline between those years stemmed mostly from two factors: sharp decreases in corporate income tax revenues during the financial crisis and the start of the severe recession, and enactment of the Economic Stimulus Act of 2008, which provided tax credits of between $300 and $1,200 to people whose income was between certain thresholds. Additional changes in tax law and slow economic growth from 2009 through 2012 kept the overall average tax rate near its lowest level over the 35-year period examined here. Primarily because many of the tax reductions in place during the 2009–2012 period expired and new higher rates went into effect, average federal tax rates jumped by almost 2 percentage points between 2012 and 2013.

Individual Income Taxes. The average individual income tax rate (individual income taxes divided by before-tax income) in 2013 was an estimated 9.2 percent, which is slightly below the average rate of 9.8 percent between 1979 and 2013. Over that period, the average individual income tax rate peaked at 11.9 percent in 1981, declined because of the reduction in tax rates enacted in 1981, and rose again after 1993 as a consequence of changes in tax law and rapidly rising incomes. After peaking again at 11.7 percent in 2000, the rate fell to 8.3 percent in 2003 because of the 2001 and 2003 tax cuts and the 2001 recession and subsequent slow recovery. The rate dropped again

Figure 12.

Average Federal Tax Rates, by Tax Source, 1979 to 2013

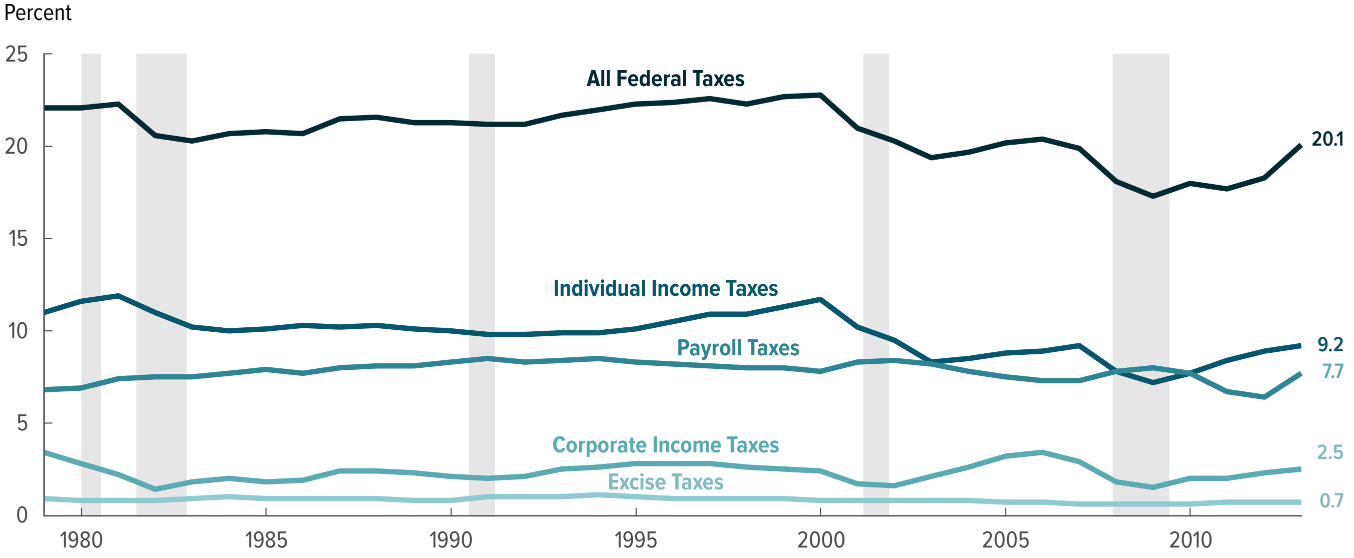

Source: Congressional Budget Office.

Average federal tax rates are calculated by dividing federal taxes by before-tax income.

Before-tax income is market income plus government transfers. Market income consists of labor income, business income, capital gains (profits realized from the sale of assets), capital income excluding capital gains, income received in retirement for past services, and other sources of income. Government transfers are cash payments and in-kind benefits from social insurance and other government assistance programs. Those transfers include payments and benefits from federal, state, and local governments.

For more detailed definitions of income, see the appendix.

in 2008 and 2009, to a low of 7.2 percent, as a result of declines in income and changes in tax law.

Between 2009 and 2013, individual income tax rates rose steadily as various tax provisions enacted during the 2007–2009 recession expired and new, higher tax rates went into effect for high-income taxpayers in 2013. Specifically, for taxpayers earning in excess of $400,000 (for individual filers) or $450,000 (for married people filing jointly), the top statutory marginal tax rate increased from 35 percent to 39.6 percent, and the tax rate for long-term capital gains and dividends increased from 15 percent to 20 percent. Furthermore, limitations on the use of personal exemptions and itemized deductions for taxpayers whose adjusted gross income was above $250,000 (for individual filers) or $300,000 (for joint filers) were reinstated in 2013. A 3.8 percent surtax was imposed on the lesser of a taxpayer's investment income and total income over certain thresholds ($200,000 for individual filers and $250,000 for joint filers) in 2013 as well.[29]

Payroll Taxes. Between 1979 and 1991, the average payroll tax rate rose steadily from 6.8 percent to a high of 8.5 percent, mostly because of legislated increases in the Social Security payroll tax rate and increases in the maximum earnings subject to that tax.[30] The payroll tax rate declined slightly in the late 1990s as labor income grew more slowly than income from other sources and as earnings above the maximum amount subject to Social Security taxes grew more rapidly than earnings below that

29. Although the tax law changes were the most significant factor driving the increase in average individual income tax rates between 2009 and 2013, at least two other factors contributed to the higher rates over that period. During the 2007–2009 recession, wage growth dipped below growth in consumer prices, which is the gauge used to adjust various parameters of the tax code for inflation. After 2009, wage growth was higher than growth in consumer prices—a return to its historical relationship. When wage growth exceeds growth in consumer prices, relatively more income falls into higher tax brackets over time—a phenomenon referred to as real bracket creep—and pushes average individual income tax rates higher. The second contributor to the increase in individual income tax rates over the 2009–2013 period was the increase in wage inequality, which also results in relatively more income being taxed at higher rates—because of the progressive structure of the individual income tax system—and thus boosting the overall average individual income tax rate.

30. The payroll tax rate is calculated as payroll taxes divided by total before-tax income. When the amount of before-tax income subject to the tax rises, the average payroll tax rate increases as well.

amount. The 2001 recession and slow recovery reversed those trends, leading the tax rate to rise a bit in the early 2000s. The rate resumed its decline in the mid-2000s when economic growth picked up, before climbing in 2008 and 2009 as the recession caused nonlabor income to fall more sharply than labor income and caused earnings above the maximum amount subject to Social Security taxes to fall more than earnings below that amount. The Tax Relief, Unemployment Insurance Reauthorization, and Job Creation Act of 2010 reduced the employee's share of Social Security payroll taxes by 2 percentage points (from 6.2 percent to 4.2 percent) for two years, which decreased the average payroll tax rate to 6.4 percent in 2012—the lowest rate observed over the 1979–2013 period. That temporary reduction in payroll taxes expired at the end of 2012. In addition, as part of the Affordable Care Act, the Medicare payroll tax rate was increased by 0.9 percentage points for taxpayers earning more than $200,000 (for individual filers) or $250,000 (for joint filers) in 2013. Those two changes pushed the average payroll tax rate up by 1.3 percentage points to 7.7 percent in 2013, which is about equal to the average rate over the 35-year period examined here.

Corporate Taxes. The average corporate income tax rate declined significantly in the early 1980s and was then fairly steady over the next two decades, with some slight shifts that generally corresponded to economic expansions and contractions. After the 2001 recession, strong growth in corporate profits pushed the corporate income tax rate up through the mid-2000s. The rate then fell by more than half between 2006 and 2009 because of the sharp drop in corporate profits during the most recent recession. As corporate profits rebounded after the recession, the average corporate income tax rate jumped by 0.5 percentage points in 2010 and rose by another 0.5 percentage points between 2011 and 2013. In 2013, the average corporate income tax rate was 2.5 percent, CBO estimates, which is slightly above the average rate between 1979 and 2013.

Excise Taxes. Unlike tax rates for the other revenue sources, the average excise tax rate was fairly stable over the entire 1979–2013 period. In 2013, the average excise tax rate was an estimated 0.7 percent.

Trends in Average Federal Tax Rates by Before-Tax Income Group

After several years of relatively low average federal tax rates, all of the income groups considered in this report experienced significant increases in 2013—especially households at the very top of the income distribution (see Figure 2 on page 4). Despite those increases in 2013, federal tax rates for most of the income distribution remained below the average rate over the 35-year period examined here. For households in the bottom four quintiles of before-tax income, the average federal tax rate in 2013 was about 3 percentage points lower than the average rate for that quintile over the 1979–2013 period, CBO estimates. For households in the 81st through 99th percentiles, the rate in 2013 was only 0.6 percentage points below the 35-year average. In contrast, the average federal tax rate in 2013 for households in the top 1 percent of the income distribution was significantly higher than the 35-year average.

Lowest Quintile. Between 1984 and 2007, the average federal tax rate for households in the lowest quintile of the distribution of before-tax income declined fairly steadily. Expansions of the earned income tax credit pushed down the average income tax rate for this group, especially in the 1990s, while increases in the average payroll tax rate partially offset that decline. Between 2007 and 2009, the average tax rate for the lowest quintile dropped markedly, largely because of provisions in the Economic Stimulus Act of 2008 and the American Recovery and Reinvestment Act of 2009 that lowered tax liabilities for low-income households. In 2009, the average federal tax rate reached its lowest point over the 35-year period—1.0 percent. The average tax rate for the lowest quintile has increased steadily since 2009, mainly because of the expiration of the Making Work Pay tax credit in 2011 and the expiration of the temporary reduction in the employee's share of the Social Security payroll tax in 2013. The average tax rate for this group in 2013—3.3 percent, CBO estimates—was about half the average rate over the entire 1979–2013 period.

Middle Three Quintiles. For most of the years between 1979 and 2013, average federal tax rates have either declined or remained relatively stable for households in this income group. The average tax rate for the middle three income quintiles fell somewhat in the early 1980s and then fluctuated within a fairly narrow range during the rest of the 1980s and the 1990s. Between 2000 and 2003, the rate declined by 3 percentage points, reflecting numerous changes in law—particularly the expansion of the child tax credit, reductions in tax rates, and increases in the standard deduction for married couples. The average tax rate for this group then edged up between 2003 and 2007 before falling by nearly 3 percentage points from 2007 to 2009. The estimated rate of 12.0 percent in 2009 was the lowest since at least 1979. Between 2009 and 2012, the average tax rate for the middle three income quintiles stayed near that low point.

That pattern of steady or decreasing average federal tax rates no longer held true in 2013. Because of changes in tax provisions between 2012 and 2013, the average federal tax rate for this group jumped by 1.4 percentage points to 13.8 percent in 2013, CBO estimates—the largest single-year increase over the 35-year period. That 2013 rate, however, is still well below the average rate over the entire period of 16.6 percent for this income group.

81st Through 99th Percentiles. The average federal tax rate for households in the 81st through 99th percentiles of the distribution of before-tax income decreased in the early 1980s and then increased a little, on balance, during the rest of that decade and in the 1990s. The rate then dropped by nearly 3 percentage points between 2000 and 2003, rose modestly from 2003 to 2007, and fell again from 2007 to 2009. Its 2009 value of 21.2 percent was the lowest since at least 1979. Between 2009 and 2012, the rate changed only slightly.

Changes in tax provisions between 2012 and 2013 caused the average federal tax rate for this group to jump by 1.5 percentage points to 23.2 percent in 2013, CBO estimates. Although that increase is the largest single-year increase for this income group over the 35-year period, the average rate in 2013 remains just below the 1979–2013 average of 23.8 percent.

Top 1 Percent. The average federal tax rate for households in the top 1 percent of the distribution of before-tax income has followed a very different pattern than the average tax rates for the other income groups. The average tax rate for this group fell sharply between 1979 and 1986, primarily because of changes in tax law, and reversed that decline between 1986 and 1995, when it peaked at 35.3 percent. The rate fell again between 1995 and 2008 and edged up, on balance, between 2008 and 2012.

In 2013, when higher individual income tax rates and new taxes enacted as part of the Affordable Care Act went into effect, average federal tax rates for this income group surged by 5.3 percentage points to 34.0 percent, CBO estimates. In anticipation of the higher tax rates going into effect in 2013, high-income taxpayers shifted some income—particularly capital gains, which are taxed at lower rates than other income sources—out of 2013 and into 2012. Because of that income shifting, the average rate in 2012 for this income group was *lower* and the average rate in 2013 was *higher* than those rates would have been without the change in tax law. In part for that reason, and in contrast to the average rates for the other income groups, the average rate in 2013 for this income group was 3.4 percentage points above the average for the 1979–2013 period.

Trends in After-Tax Income

Changes in economic conditions, government transfer programs, and federal tax laws have resulted in after-tax income growing at different rates across the income spectrum over time. For households in the lowest quintile of before-tax income, inflation-adjusted after-tax income was 46 percent higher in 2013 than it was in 1979, CBO estimates, slightly greater than the 41 percent increase for households in the 21st to 80th percentiles. Cumulative growth in the inflation-adjusted after-tax income of households in the 81st to 99th percentiles and the top 1 percent of the before-tax income distribution was substantially greater, an estimated 70 percent and 192 percent, respectively (see Figure 13). Those cumulative growth figures are equivalent to average annual growth rates over the 35-year period of 1.1 percent, 1.0 percent, 1.6 percent, and 3.2 percent, respectively. Each of those cumulative percentage increases in after-tax income exceeds the cumulative percentage increases in before-tax income for the corresponding income groups, because the average federal tax rate for each income group was lower in 2013 than in 1979.

Trends in Income Inequality

As the distributions of income have shifted over time, so has the degree of inequality in market income, before-tax income, and after-tax income.[31] A standard measure

31. A significant body of research has examined changes in U.S. income inequality over time, using various data sources and measures of income. For example, Thomas Piketty and Emmanuel Saez rely on income tax data from the Internal Revenue Service's Statistics of Income; see Piketty and Saez, "Income Inequality in the United States, 1913–1998," *Quarterly Journal of Economics*, vol. 118, no. 1 (February 2003), pp. 1–39, http://eml.berkeley.edu/~saez/pikettyqje.pdf (471 KB). Timothy M. Smeeding and Jeffrey P. Thompson rely on the Federal Reserve's Survey of Consumer Finances; see Smeeding and Thompson, "Recent Trends in Income Inequality," in Herwig Immervoll, Andreas Peichl, and Konstantinos Tatsiramos, eds., *Who Loses in the Downturn? Economic Crisis, Employment, and Income Distribution,* vol. 32 of *Research in Labor Economics* (Emerald Group Publishing), pp.1–50, http://tinyurl.com/qdk92x4. Philip Armour, Richard V. Burkhauser, and Jeff Larrimore rely on the Census Bureau's Current Population Survey; see Armour, Burkhauser, and Larrimore, *Levels and Trends in United States Income and Its Distribution: A Crosswalk From Market Income Towards a Comprehensive Haig-Simons Income Approach*, Working Paper 19110 (National Bureau of Economic Research, June 2013), www.nber.org/papers/w19110.

Figure 13.

Cumulative Growth in Average Inflation-Adjusted After-Tax Income, by Before-Tax Income Group, 1979 to 2013

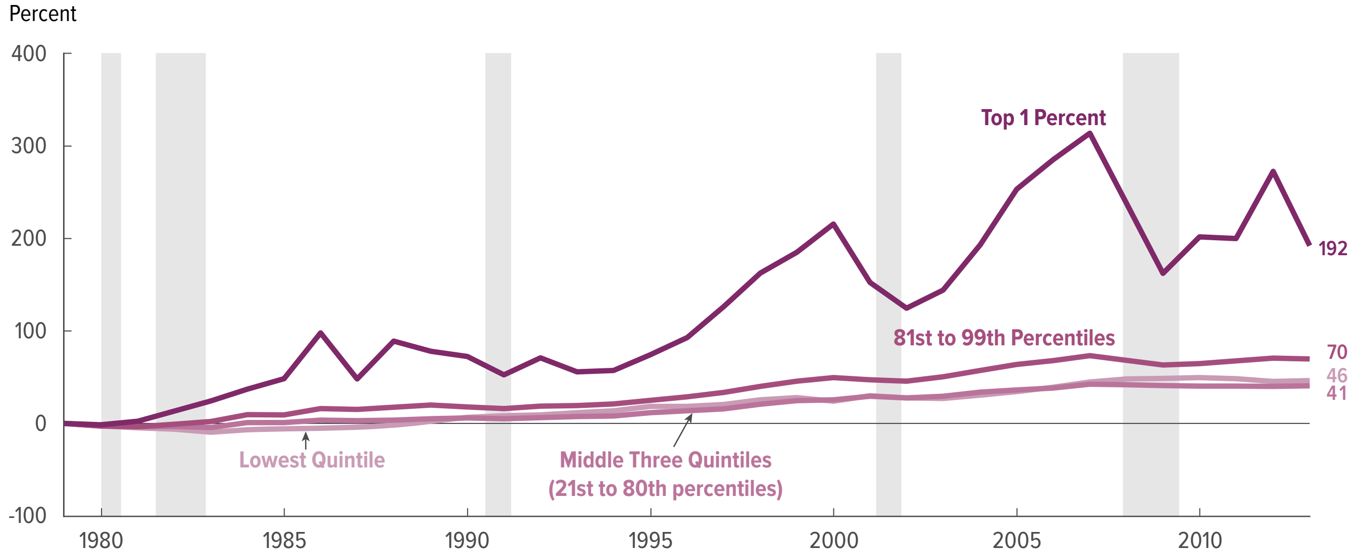

Source: Congressional Budget Office.

After-tax income is before-tax income minus federal taxes.

Before-tax income is market income plus government transfers. Market income consists of labor income, business income, capital gains (profits realized from the sale of assets), capital income excluding capital gains, income received in retirement for past services, and other sources of income. Government transfers are cash payments and in-kind benefits from social insurance and other government assistance programs. Those transfers include payments and benefits from federal, state, and local governments.

Federal taxes include individual income taxes, payroll taxes, corporate income taxes, and excise taxes.

Income is converted to 2013 dollars using the price index for personal consumption expenditures.

Income groups are created by ranking households by before-tax income, adjusted for household size. Quintiles (fifths) contain equal numbers of people; percentiles (hundredths) contain equal numbers of people as well.

For more detailed definitions of income, see the appendix.

of income inequality is the Gini index, which summarizes an entire distribution in a single number that ranges from zero to one. A value of zero means that income is distributed equally among all income groups, and a value of one indicates that all of the income is received by the highest-income group and none is received by any of the lower-income groups. Inequality of all three measures of income was higher in 2013 than in 1979, according to CBO's estimates (see Figure 14).[32]

The increase in inequality in both before-tax and after-tax income over the 35-year period stemmed largely from a significant increase in inequality in market income, mostly because of substantial income growth at the top of the market income distribution. In 1979, the Gini index for market income was 0.48. In 2013, the Gini index was 0.60, or approximately 25 percent higher than the index in 1979.

Inequality as measured by the Gini index has been consistently lower for after-tax income than for before-tax income and consistently lower for before-tax income than for market income.[33] Government transfers reduce income inequality because the transfers received by lower-income households are larger relative to their market income than are the transfers received by higher-income households. Federal taxes also reduce income inequality, because the taxes paid by higher-income households are larger relative to their before-tax income than are the taxes paid by lower-income households. The equalizing effects of government transfers were significantly larger than the equalizing effects of federal taxes from 1979 to 2013.

32. Gini indexes in this report are calculated using incomes adjusted for household size. For a more detailed discussion of CBO's approach to calculating Gini indexes, see *Trends in the Distribution of Household Income Between 1979 and 2007* (October 2011), www.cbo.gov/publication/42729.

33. Gini indexes for market, before-tax, and after-tax income reflect rankings of households based on those income measures. Therefore, some of the differences between the Gini indexes are attributable to the reranking of households across the indexes.

Figure 14.

Gini Indexes Based on Market, Before-Tax, and After-Tax Income, 1979 to 2013

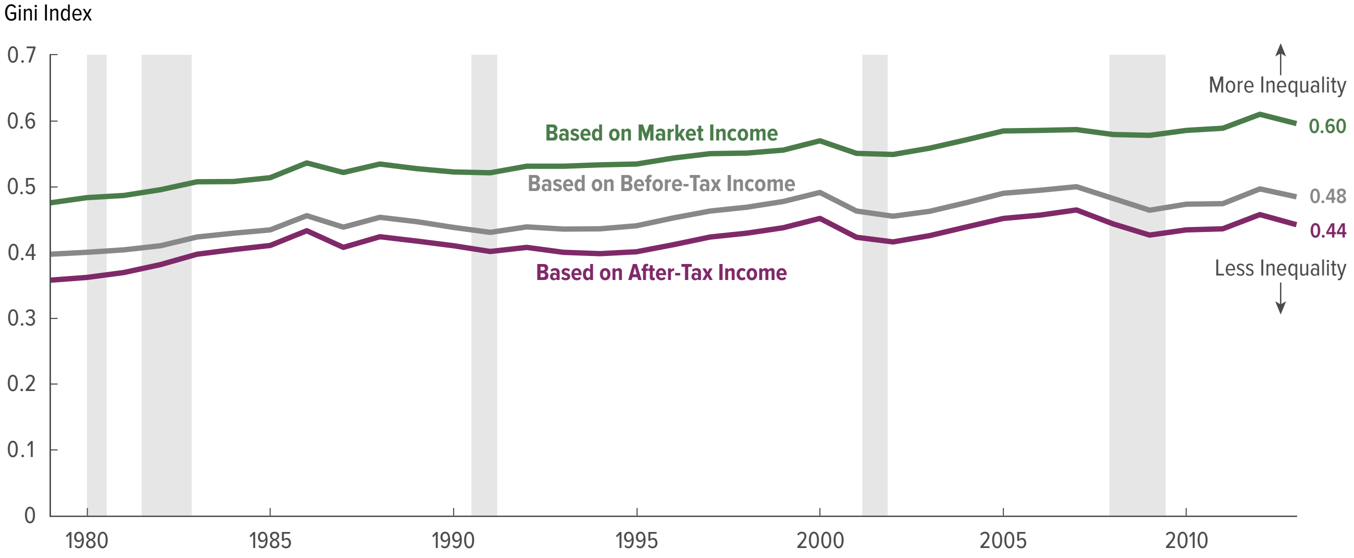

Source: Congressional Budget Office.

The Gini index is a measure of income inequality that ranges from zero (the most equal distribution) to one (the least equal distribution). Gini indexes are calculated using income measures adjusted for household size.

Market income consists of labor income, business income, capital gains (profits realized from the sale of assets), capital income excluding capital gains, income received in retirement for past services, and other sources of income.

Before-tax income is market income plus government transfers. Government transfers are cash payments and in-kind benefits from social insurance and other government assistance programs. Those transfers include payments and benefits from federal, state, and local governments.

After-tax income is before-tax income minus federal taxes. Federal taxes include individual income taxes, payroll taxes, corporate income taxes, and excise taxes.

For more detailed definitions of income, see the appendix.

The effect of federal taxes on income inequality was quite stable between the mid-1990s and 2013 because of two trends that were roughly offsetting (see Figure 15). First, the share of federal taxes paid by higher-income households increased more than the share of income earned by those households. For example, the share of before-tax income received by households in the top quintile increased by about 3 percentage points between 1995 and 2013, whereas the share of federal taxes paid by those households increased by about 8 percentage points over that period, CBO estimates. Second, average federal tax rates across the income spectrum decreased. Because federal taxes became both more progressive and smaller relative to before-tax income between the mid-1990s and 2013, those taxes had roughly the same effect in reducing income inequality throughout that period.

Despite the relative stability of the equalizing effects of the federal tax system, small decreases in income inequality are apparent during the past two recessions as the share of labor income increased while the share of capital income (which is subject to lower, less progressive tax rates than labor income) fell. A small decrease in income inequality resulting from changes in the federal tax system is also apparent in the data for 2013. That decrease in income inequality stemmed primarily from the higher rates faced by high-income taxpayers in that year, which made the federal tax system the most progressive it has been since the mid-1990s.[34]

34. There are several ways to measure the progressivity of the federal tax system. One such measure, known as the Reynolds-Smolensky index, is equal to the difference between the Gini index for before-tax income and the Gini index for after-tax income. Another measure of the progressivity of the federal tax system, known as the Kakwani index, is computed as the difference between the concentration of tax payments (with households ranked by their before-tax household income) and the Gini index for before-tax income. Those alternative measures of progressivity are available in the supplemental data posted along with this report on CBO's website (www.cbo.gov/publication/51361). For more detailed information on those progressivity measures, see *Trends in the Distribution of Household Income Between 1979 and 2007* (October 2011), www.cbo.gov/publication/42729.

Figure 15.

Reduction in Income Inequality From Government Transfers and Federal Taxes, 1979 to 2013

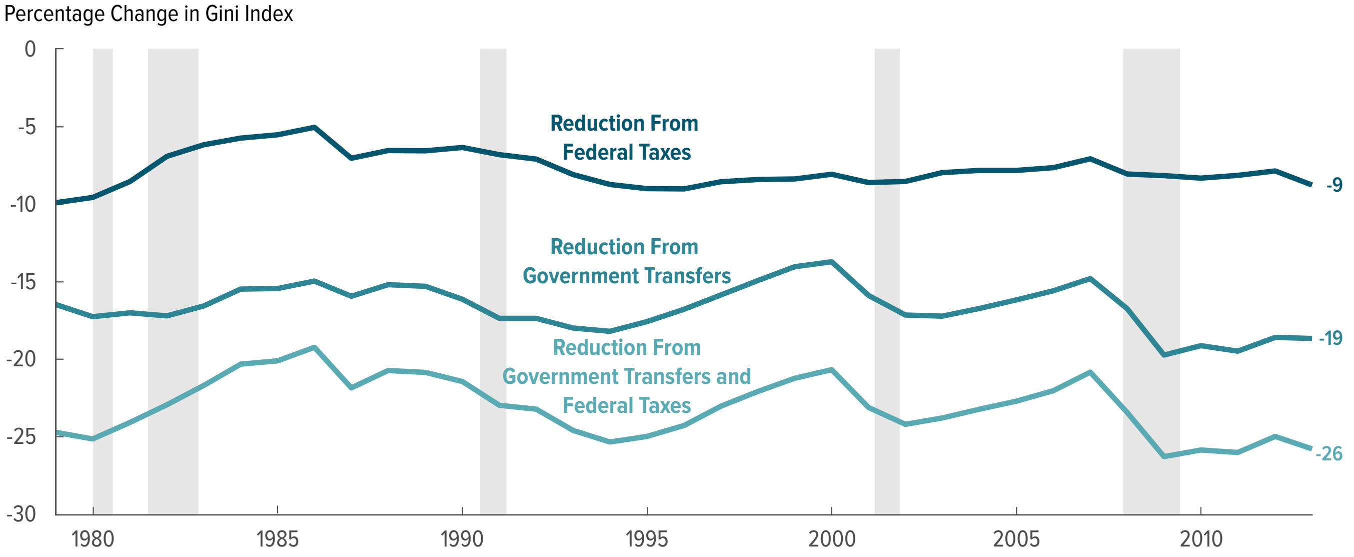

Source: Congressional Budget Office.

The Gini index is a measure of income inequality that ranges from zero (the most equal distribution) to one (the least equal distribution). Gini indexes are calculated using income measures adjusted for household size.

Government transfers are cash payments and in-kind benefits from social insurance and other government assistance programs. Those transfers include payments and benefits from federal, state, and local governments.

Federal taxes include individual income taxes, payroll taxes, corporate income taxes, and excise taxes.

The effect of government transfers on income inequality varies across economic expansions and contractions because such transfers tend to decline when the economy is strong and to increase when the economy is weak—because of both automatic changes in benefits from some programs (such as unemployment insurance) and legislated changes in response to fluctuating economic conditions. During the most recent recession, the percentage decline in the Gini index attributable to government transfers increased from 15 percent in 2007 to 20 percent in 2009, CBO estimates.[35] That additional reduction in inequality is attributable primarily to an automatic increase in unemployment insurance benefits and significant legislated expansions of those benefits and benefits from the Supplemental Nutrition Assistance Program.[36] Between 2009 and 2013, the equalizing effect of government transfers diminished slightly; although the expansions in unemployment insurance and SNAP benefits remained in place through all or most of 2013, respectively, the unemployment rate gradually fell over that period and the number of households receiving the expanded unemployment benefits decreased.[37]

35. Transfers as measured in this report do not equal total government spending on the transfers because some transfers go to recipients outside the scope of the survey data collected by the Census Bureau and because recipients underreport, on average, the amount of transfer payments they receive. Therefore, the percentage reduction in the Gini index attributable to transfers probably understates the true magnitude of the effect.

36. See Congressional Budget Office, *Unemployment Insurance in the Wake of the Recent Recession* (November 2012), www.cbo.gov/publication/43734, and *The Supplemental Nutrition Assistance Program* (April 2012), www.cbo.gov/publication/43173.

37. The duration of unemployment benefits available to unemployed workers was originally expanded as part of the Supplemental Appropriations Act of 2008 and was modified several times thereafter. The American Taxpayer Relief Act of 2012 extended those expanded benefits through the end of 2013. SNAP benefits were originally expanded as part of the American Recovery and Reinvestment Act of 2009. Those expanded benefits expired on October 31, 2013.

Appendix: Data and Methods

In this report, the Congressional Budget Office examines the distribution of household income and federal tax rates, using data for calendar year 2013, the most recent data available with complete information about tax payments. The current analysis builds on earlier CBO analyses, the most recent of which covered the period from 1979 through 2011.[1]

Data Sources on Household Income

Information on household income for this analysis came from two primary sources: the Internal Revenue Service's Statistics of Income (SOI) and the Census Bureau's Current Population Survey (CPS). The core data came from the SOI, a nationally representative sample of individual income tax returns. The number of returns sampled grew over the time period studied, ranging from roughly 90,000 in some of the early years to more than 300,000 in the later years. CBO used the full Individual Income Tax file, which is more detailed than the public-use version of the file. The agency supplemented those data with data from the CPS's Annual Social and Economic Supplement; those data identify demographic characteristics and income for a large sample of households.

Both the SOI and the CPS lack important information needed for estimating and comparing after-tax household income over time. The SOI does not include information about people who do not file federal tax returns, and it does not report all income from government cash transfer programs. It also offers no information on the receipt of in-kind transfers and benefits, and it is organized by tax filing units rather than by households. The CPS lacks detailed information on high-income households, it does not report capital gains, it underreports other income from capital, and it lacks information on the deductions and adjustments necessary to compute taxes.

Together, however, the two sources can form a more complete picture. To overcome limitations in each source of data, CBO statistically matched SOI records to corresponding CPS records on the basis of demographic characteristics and income. Each pairing resulted in a new record that took on the demographic characteristics of the CPS record and the income reported in the SOI. (Some types of income—certain transfers and in-kind benefits, for example—appear only in the CPS; their values were drawn directly from that survey.) Because some households are not required to file tax returns, the SOI does not have data on every household in the nation. To create a sample that was representative of the entire population, once all SOI records were matched to CPS records, the remaining CPS records were recorded as nonfiling households and their income values were taken directly from the CPS.

Because of the significant effort required to collect and process the SOI and the CPS data, there is a lag between when the data become available and the time frame covered by the data. For the CPS's Annual Social and Economic Supplement, the Census Bureau collects data from households each spring covering the previous calendar year. The processing then takes several months, so the data generally are available in the fall. The CPS data for calendar year 2013, for example, became available in the fall of 2014. Because tax returns for a given calendar year are filed throughout the following year, there is a more significant lag in the SOI data. Those data are generally released each summer, but with a two-year lag. The SOI data for tax year 2013, for example, became available during the summer of 2015. To produce more timely information on the distribution of

1. CBO's reports on household income and taxation are posted together on the agency's website. See Congressional Budget Office, "Major Recurring Reports: Distribution of Household Income and Federal Taxes," www.cbo.gov/about/our-products/RecurringReports#15. Supplemental data that present estimates for the 1979–2013 period are available at www.cbo.gov/publication/51361.

household income and federal taxes, CBO is developing the capacity to project those distributions beyond the last year available in the data.

Allocation of Taxes to Households

CBO allocated individual income taxes and the employee's share of payroll taxes to the households paying those taxes directly.[2] The agency also allocated the employer's share of payroll taxes to employees because employers appear to pass on their share of payroll taxes to employees by paying lower wages than they would otherwise pay.[3] Therefore, CBO also added the employer's share of payroll taxes to households' earnings when calculating before-tax income.

CBO allocated excise taxes to households according to their consumption of taxed goods and services (such as gasoline, tobacco, and alcohol). Excise taxes on intermediate goods, which are paid by businesses, were attributed to households in proportion to their overall consumption. CBO assumed that each household's spending on taxed goods and services was the same as that reported in the Bureau of Labor Statistics' Consumer Expenditure Survey for a household with comparable income and other characteristics.

Far less consensus exists about how to allocate corporate income taxes (and taxes on capital income generally). In this analysis, CBO allocated 75 percent of corporate income taxes to owners of capital in proportion to their income from interest, dividends, rents, and adjusted capital gains; the adjustment smooths out large year-to-year variations in actual realized gains by scaling them to an estimate of their long-term historical level given the size of the economy and the tax rate that applies to them.[4] CBO allocated the remaining 25 percent of corporate income taxes to workers in proportion to their labor income.

Measures of Income

This analysis uses three measures of household income: market income, market income plus government transfers (before-tax income), and market income plus government transfers minus federal taxes (after-tax income).

Market income consists of these components:

- *Labor Income.* Cash wages and salaries, including amounts allocated by employees to 401(k) plans; employer-paid health insurance premiums; the employer's share of Social Security, Medicare, and federal unemployment insurance payroll taxes; and the share of corporate income taxes borne by workers.
- *Business Income.* Net income from businesses and farms operated solely by their owners, partnership income, and income from S corporations.
- *Capital Gains.* Profits realized from the sale of assets, excluding increases in the value of assets that have not been realized through sales.[5]
- *Capital Income Excluding Capital Gains.* Taxable and tax-exempt interest, dividends paid by corporations (but not dividends paid by S corporations, which are

2. For this analysis, CBO considered refundable tax credits as negative income taxes rather than transfers. Federal taxes in this analysis were incurred in 2013 but not necessarily paid to the government in that year.

3. That allocation reflects a consensus among economists. See Don Fullerton and Gilbert E. Metcalf, "Tax Incidence," in Alan J. Auerbach and Martin Feldstein, eds., *Handbook of Public Economics*, vol. 4 (Elsevier, 2002), pp. 1787–1872.

4. For a more detailed discussion of CBO's methodology for allocating corporate taxes, see Congressional Budget Office, *The Distribution of Household Income and Federal Taxes, 2008 and 2009* (July 2012), www.cbo.gov/publication/43373.

5. CBO measures income from capital gains when realized, partly because estimates of accrued gains depend crucially on assumptions that are very uncertain and partly because maintaining consistency with the measurement of taxes (which are levied on realized gains) is valuable. Various researchers have measured capital gains as they accrue, on the basis of assets held by households at different income levels; those researchers have reached disparate conclusions about the effect of accrued capital gains on levels and trends in income inequality, depending in large part on their assumptions about the rate of return on assets. See, for example, Timothy M. Smeeding and Jeffrey P. Thompson, "Recent Trends in Income Inequality," in Herwig Immervoll, Andreas Peichl, and Konstantinos Tatsiramos, eds., *Who Loses in the Downturn? Economic Crisis, Employment, and Income Distribution,* vol. 32 of *Research in Labor Economics* (Emerald Group Publishing), pp. 1–50, http://tinyurl.com/qdk92x4; and Philip Armour, Richard V. Burkhauser, and Jeff Larrimore, *Levels and Trends in United States Income and Its Distribution: A Crosswalk From Market Income Towards a Comprehensive Haig-Simons Income Approach*, Working Paper 19110 (National Bureau of Economic Research, June 2013), www.nber.org/papers/w19110.

considered part of business income), positive rental income, and the share of corporate income taxes borne by owners of capital.

- *Other Income.* Income received in retirement for past services or from other sources.[6]

Government transfers consist of the cost of two types of benefits:

- *Cash.* Payments from Social Security, unemployment insurance, Supplemental Security Income, Temporary Assistance for Needy Families (and its predecessor, Aid to Families With Dependent Children), veterans' programs, workers' compensation, and state and local government assistance programs.

- *In-Kind Benefits.* The cost of Supplemental Nutrition Assistance Program vouchers (popularly known as food stamps); school lunches and breakfasts; housing assistance; energy assistance; and benefits provided by Medicare, Medicaid, and the Children's Health Insurance Program.[7]

This report focuses on the distribution of annual income. Other analysts and government agencies that study the distribution of income use a variety of data sources, use different units of analysis, and include different components of income in the construction of their measures of income.[8]

The data used in this analysis contain information on separate samples of households for each year. Therefore, it is not possible to link households across years, and income groups in each year contain a different set of households. Furthermore, this report does not assess trends in the distribution of other measures of economic well-being, such as household income measured over longer periods, household consumption, or household wealth.[9]

Adjusting Income for Differences Among Households

CBO used the household as the unit of analysis for this report. A household consists of the people who share a housing unit, regardless of their relationship. Analyzing income and taxes on a household basis is most useful if households make joint economic decisions, which is probably true in most cases but not all (such as group houses). A household can consist of more than one tax-paying unit, such as a married couple living with an adult child.

Households with identical income can differ in ways that affect their economic status. A larger household generally needs more income to support a given standard of living than a smaller one does. However, economies of scale in some types of consumption—housing, in particular—can mean that two people generally do not need twice the income to live as well as one person who lives alone. Therefore, to rank households by their standard of living, it is probably appropriate to divide household income by an adjustment factor that is between one (which would result only in household income and would not capture the greater needs of larger households) and the number of people in the household (which would produce household income per person and would not capture the benefits of shared consumption). CBO chose to adjust for household size by dividing household income by the square root of the number of people in the household, counting adults and children equally.[10] Households were then ranked by adjusted income and grouped into quintiles (or fifths) of equal numbers of people. Because

6. Most of the income in this category is pension income. That income includes taxable annuity payments from defined benefit pension plans; taxable withdrawals from defined contribution plans, such as 401(k)s; and taxable withdrawals from individual retirement accounts.

7. Health care benefits are measured using the Census Bureau's estimates of the average cost to the government of providing those benefits. For more details on CBO's approach to valuing health care benefits and the consequences of that approach, see Congressional Budget Office, *The Distribution of Household Income and Federal Taxes, 2008 and 2009* (July 2012), www.cbo.gov/publication/43373.

8. For a summary of the sources included in the income measures used by others conducting distributional analyses, see Dennis Fixler and David S. Johnson, "Accounting for the Distribution of Income in the U.S. National Accounts" in Dale W. Jorgenson, J. Steven Landefeld, and Paul Schreyer, eds., *Measuring Economic Stability and Progress*, vol. 72 in *Research Studies in Income and Wealth* (National Bureau of Economic Research, 2014), pp. 213–244, www.nber.org/chapters/c12828.

9. Income measured over several years is more equally distributed—although only modestly so—than income for a single year. See Congressional Budget Office, *Effective Tax Rates: Comparing Annual and Multiyear Measures* (January 2005), www.cbo.gov/publication/16212.

household sizes vary, different quintiles generally have slightly different numbers of households.

Adjusting income for other differences in circumstances that affect a household's standard of living also could be desirable. For example, the prices of goods and services vary geographically, and households can incur different costs associated with working—for commuting and child care expenses, for example—depending on how many members are employed. CBO did not adjust for any such additional differences.

Types of Households

Changes in the composition of household types—and household sizes—can affect the distribution of income over time. Supplemental tables, available online, show household income and average federal tax rates for three types of households: those with members under age 18 (households with children), those headed by a person age 65 or older and with no members under age 18 (elderly childless households), and all others (nonelderly childless households). The tables group households into quintiles by their position in the income distribution across the full population, not by their position in the income distribution for each type of household.

10. That adjustment implies that each additional person increases a household's needs but does so at a decreasing rate. For example, a household consisting of a married couple with two children and an income of $80,000 would have an adjusted income of $40,000 ($\$80{,}000/\sqrt{4}$) and would have the equivalent economic ranking of a single person with an income of $40,000 or of a childless married couple with an income of $56,600 ($\$56{,}600/\sqrt{2}$ is almost $40,000). See Constance F. Citro and Robert T. Michael, eds., *Measuring Poverty: A New Approach* (National Research Council, 1995), http://tinyurl.com/mdhqjb4.

List of Tables and Figures

Tables

1. Average Household Income, Transfers, and Taxes, by Before-Tax Income Group, 2013 2
2. Components of Average Market Income, by Market Income Group, 2013 7
3. Components of Average Before-Tax Income, by Before-Tax Income Group, 2013 8

Figures

1. Shares of Before-Tax Income and Federal Taxes, by Before-Tax Income Group, 2013 3
2. Average Federal Tax Rates, by Before-Tax Income Group, 1979 to 2013 4
3. Average Market Income, by Market Income Group, 2013 6
4. Average Federal Tax Rates, by Before-Tax Income Group, 2013 11
5. Average Federal Tax Rates, by Before-Tax Income Group and Tax Source, 2013 12
6. Shares of Before-Tax and After-Tax Income, by Before-Tax Income Group, 2013 13
7. Shares of Market and After-Tax Income, by Market Income Group, 2013 14
8. Cumulative Growth in Average Inflation-Adjusted Market Income, by Market Income Group, 1979 to 2013 15
9. Components of Inflation-Adjusted Market Income for the Top 1 Percent of Households, 1979 to 2013 16
10. Shares of Market Income Excluding Capital Gains, by Source, for the Top 1 Percent of Households, 1979 to 2013 17
11. Cumulative Growth in Average Inflation-Adjusted Before-Tax Income, by Before-Tax Income Group, 1979 to 2013 18
12. Average Federal Tax Rates, by Tax Source, 1979 to 2013 19
13. Cumulative Growth in Average Inflation-Adjusted After-Tax Income, by Before-Tax Income Group, 1979 to 2013 22
14. Gini Indexes Based on Market, Before-Tax, and After-Tax Income, 1979 to 2013 23
15. Reduction in Income Inequality From Government Transfers and Federal Taxes, 1979 to 2013 24

About This Document

This Congressional Budget Office report was prepared at the request of the Ranking Member of the Senate Finance Committee. In keeping with CBO's mandate to provide objective, impartial analysis, the report makes no recommendations.

Kevin Perese of CBO's Tax Analysis Division wrote the report, with guidance from Edward Harris and David Weiner. Naveen Singhal provided helpful comments. Jeffrey Kling and Robert Sunshine reviewed the report, Christine Bogusz edited it, and Maureen Costantino and Jeanine Rees prepared it for publication. An electronic version is available on CBO's website (www.cbo.gov/publication/51361).

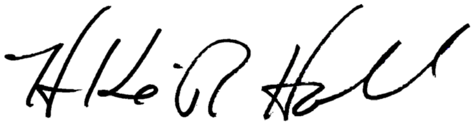

Keith Hall
Director

June 2016
