# Supplementary material for: Victory Tax: A Holistic Income Tax System
Source: Entropy (Basel). 2021 Nov 11;23(11):1492. doi: 10.3390/e23111492 (PMC8624733; doi:10.3390/e23111492)
Supplement: Supplementary file 1 [file entropy-23-01492-s001.zip › bundle/results_to_give_away/supporting_gov_data/2009HHSPovertyGuidelinesASPE.pdf]

U.S. Department of Health & Human Services

ASPE

OFFICE OF THE ASSISTANT SECRETARY
FOR PLANNING AND EVALUATION

2009 HHS POVERTY GUIDELINES

12/01/2009

THE 2009 HHS POVERTY GUIDEL

ONE VERSION OF THE [U.S.] FEDERAL POVERT

[Latest Poverty Guidelines]
[*Federal Register* Notice, January 23, 2009 — Full text]
[Prior Poverty Guidelines and *Federal Register* References Since
[Frequently Asked Questions (FAQs)]
[Further Resources on Poverty Measurement, Poverty Lines, and Thei
[Computations for the 2009 Poverty Guidelines]

There are two slightly different versions of the federal poverty measure:

- The poverty thresholds, and
- The poverty guidelines.

The **poverty thresholds** are the original version of the federal poverty measure. They are updated each year by the **Cens** Orshansky of the Social Security Administration). The thresholds are used mainly for **statistical** purposes — for instance, (In other words, all official poverty population figures are calculated using the poverty thresholds, not the guidelines.) Pove average poverty thresholds since 1959 are available on the Census Bureau's Web site. For an example of how the Census poverty status, see "How the Census Bureau Measures Poverty" on the Census Bureau's web site.

The **poverty guidelines** are the other version of the federal poverty measure. They are issued each year in the *Federal Re* The guidelines are a simplification of the poverty thresholds for use for **administrative** purposes — for instance, determinir *Register* notice of the 2009 poverty guidelines is available.

The poverty guidelines are sometimes loosely referred to as the "federal poverty level" (FPL), but that phrase is ambiguous administrative) where precision is important.

Key differences between poverty thresholds and poverty guidelines are outlined in a table under Frequently Asked Questior Research on Poverty's web site.

NOTE: The poverty guideline figures below are NOT the figures the Census Bureau uses to ca

The figures that the Census Bureau uses are the poverty thres

The 2009 Poverty Guidelines for the 48 Contiguous States and the District of Colu

PERSONS IN FAMILY	POVERTY GUIDELINE
1	$10,830
2	14,570
3	18,310
4	22,050
5	25,790
6	29,530
7	33,270
8	37,010
For families with more than 8 persons, add $3,740 for each additional person.	

2009 Poverty Guidelines for

2009 Poverty Guidelines for Alaska

PERSONS IN FAMILY	POVERTY GUIDELINE
1	$13,530
2	18,210
3	22,890
4	27,570
5	32,250
6	36,930
7	41,610
8	46,290
For families with more than 8 persons, add $4,680 for each additional person.	

2009 Poverty Guidelines for Hawaii

PERSONS IN FAMILY	POVERTY GUIDELINE
1	$12,460
2	16,760
3	21,060
4	25,360
5	29,660
6	33,960
7	38,260

8	42,560
For families with more than 8 persons, add $4,300 for each additional person.	

SOURCE: *Federal Register*, Vol. 74, No. 14, January 23, 2009, p

The separate poverty guidelines for Alaska and Hawaii reflect Office of Economic Opportunity administrative practice begin original version of the poverty measure — have never had separate figures for Alaska and Hawaii. The poverty guidelines Samoa, Guam, the Republic of the Marshall Islands, the Federated States of Micronesia, the Commonwealth of the Northe using the poverty guidelines serves any of those jurisdictions, the Federal office which administers the program is responsil guidelines for those jurisdictions or to follow some other procedure.

The poverty guidelines apply to both aged and non-aged units. The guidelines have never had an aged/non-aged distinctic separate figures for aged and non-aged one-person and two-person units.

Programs using the guidelines (or percentage multiples of the guidelines — for instance, 125 percent or 185 percent of the Stamp Program, the National School Lunch Program, the Low-Income Home Energy Assistance Program, and the Childrer assistance programs (Temporary Assistance for Needy Families and Supplemental Security Income) do NOT use the pove program also does NOT use the poverty guidelines to determine eligibility. For a more detailed list of programs that do and

The poverty guidelines (unlike the poverty thresholds) are designated by the year in which they are issued. For instance, tl guidelines. However, the 2009 HHS poverty guidelines only reflect price changes through calendar year 2008; accordingly, thresholds for calendar year 2008. (The 2008 thresholds are expected to be issued in final form in August 2009; a prelimin Bureau.)

The computations for the 2009 poverty guidelines are available.

The poverty guidelines may be formally referenced as "the poverty guidelines updated periodically in the

by the U.S. Department of Health and Human Services under the authority of 42 U.S.C. 9902(2)."

Was this page helpful? *

Yes No

NEXT >

Form Approved OMB# 0990-0379 Exp. Date 8/31/2017

osaspeinfo@hhs.gov
