# Supplementary material for: Victory Tax: A Holistic Income Tax System
Source: Entropy (Basel). 2021 Nov 11;23(11):1492. doi: 10.3390/e23111492 (PMC8624733; doi:10.3390/e23111492)
Supplement: Supplementary file 1 [file entropy-23-01492-s001.zip › bundle/results_to_give_away/supporting_gov_data/government transfer data.pdf]

Table 539. Government Transfer Payments to Individuals—Summary: 1990 to 2009

[In billions of dollars (566.1 represents $566,100,000,000)]

Year	Transfer payments, total	Retirement and disability insurance benefits	Medical payments	Income maintenance benefits	Unemployment insurance benefits	Veterans benefits	Federal education and training assistance payments [1]	Other [2]
1990	566.1	263.9	188.8	63.5	18.2	17.7	12.3	1.7
1995	849.8	350.0	338.6	100.4	21.8	20.5	17.2	1.2
1998	940.9	391.2	383.4	101.1	19.9	23.3	20.5	1.6
1999	975.7	402.4	400.7	104.8	20.7	24.1	21.3	1.7
2000	1,027.8	424.5	427.2	106.3	21.0	25.0	21.9	2.0
2001	1,126.7	449.8	480.8	109.4	32.1	26.6	25.4	2.6
2002	1,232.1	474.4	523.8	120.7	53.7	29.5	27.9	2.1
2003	1,299.0	493.5	555.8	133.2	53.6	31.8	28.5	2.7
2004	1,381.2	517.1	610.3	144.2	37.1	34.0	31.2	7.3
2005	1,465.1	545.4	653.2	159.6	32.3	36.4	33.8	4.5
2006	1,565.6	576.9	717.0	163.4	30.9	38.9	35.9	2.7
2007	1,669.8	609.1	771.4	172.3	33.4	41.7	39.5	2.6
2008	1,824.1	639.6	822.1	185.8	52.0	45.1	45.6	33.8
2009	2,076.1	699.5	892.4	217.9	130.1	51.4	56.7	28.0

[1] See footnote 9, Table 540. [2] See footnote 10, Table 540.

Source: U.S. Bureau of Economic Analysis, "Regional Accounts Data, Annual State Personal Income," <http://www.bea.gov/regional/spi/>, accessed March 2011.

Table 540. Government Transfer Payments to Individuals by Type: 1990 to 2009

[In millions of dollars (566,100 represents $566,100,000,000)]

Item	1990	2000	2005	2006	2007	2008	2009
**Total**	**566,100**	**1,027,827**	**1,465,125**	**1,565,646**	**1,669,795**	**1,824,122**	**2,076,109**
Retirement & disability insurance benefit payments	263,888	424,461	545,361	576,904	609,100	639,619	699,519
Old-age, survivors, & disability insurance	244,135	401,393	512,728	544,096	575,648	605,530	664,287
Railroad retirement and disability	7,221	8,267	9,191	9,519	9,813	10,068	10,630
Workers' compensation payments (federal & state)	8,618	10,898	15,866	15,650	15,794	16,120	16,434
Other government disability insurance & retirement [1]	3,914	3,903	7,576	7,639	7,845	7,901	8,168
Medical payments	188,808	427,194	653,193	717,010	771,361	822,111	892,410
Medicare	107,638	219,117	331,924	399,193	427,556	462,773	500,254
Public assistance medical care [2]	78,176	205,021	315,032	310,977	336,884	351,846	383,614
Military medical insurance [3]	2,994	3,056	6,237	6,840	6,921	7,492	8,542
Income maintenance benefit payments	63,481	106,285	159,624	163,418	172,255	185,846	217,858
Supplemental Security Income (SSI)	16,670	31,675	38,285	39,892	42,285	44,150	47,534
Family assistance [4]	19,187	18,440	18,216	18,226	18,334	19,255	20,088
Food stamps	14,741	14,565	29,492	29,390	30,920	36,987	54,574
Other income maintenance [5]	12,883	41,605	73,631	75,910	80,716	85,454	95,662
Unemployment insurance benefit payments	18,208	20,989	32,276	30,900	33,381	52,022	130,141
State unemployment insurance compensation	17,644	20,223	31,001	29,594	32,006	50,318	127,915
Unemployment compensation for federal civilian employees	215	226	224	218	216	255	439
Unemployment compensation for railroad employees	89	81	72	78	83	84	179
Unemployment compensation for veterans	144	181	446	449	406	467	840
Other unemployment compensation [6]	116	278	533	561	670	898	768
Veterans benefit payments	17,687	25,004	36,371	38,877	41,676	45,125	51,429
Veterans pension and disability	15,550	21,966	32,505	35,018	37,721	40,798	45,391
Veterans readjustment [7]	257	1,322	2,256	2,290	2,421	2,783	4,518
Veterans life insurance benefits	1,868	1,706	1,596	1,554	1,517	1,523	1,493
Other assistance to veterans [8]	12	10	14	15	17	21	27
Federal education & training assistance payments [9]	12,286	21,851	33,796	35,859	39,450	45,577	56,747
Other payments to individuals [10]	1,742	2,043	4,504	2,678	2,572	33,822	28,005

[1] Consists largely of temporary disability payments, pension benefit guaranty payments, and black lung payments. [2] Consists of medicaid and other medical vendor payments. [3] Consists of payments made under the TriCare Management Program (formerly called CHAMPUS) for the medical care of dependents of active duty military personnel and of retired military personnel and their dependents at nonmilitary medical facilities. [4] Through 1990, consists of emergency assistance and aid to families with dependent children. Beginning with 2000, consists of benefits—generally known as temporary assistance for needy families—provided under the Personal Responsibility and Work Opportunity Reconciliation Act of 1996. [5] Consists largely of general assistance, expenditures for food under the supplemental program for women, infants, and children; refugee assistance; foster home care and adoption assistance; earned income tax credits; and energy assistance. [6] Consists of trade readjustment allowance payments, Redwood Park benefit payments, public service employment benefit payments, and transitional benefit payments. [7] Consists largely of veterans' readjustment benefit payments, educational assistance to spouses and children of disabled or deceased veterans, payments to paraplegics, and payments for autos and conveyances for disabled veterans. [8] Consists largely of state and local government payments to veterans. [9] Excludes veterans. Consists largely of federal fellowship payments (National Science Foundation fellowships and traineeships, subsistence payments to state maritime academy cadets, and other federal fellowships), interest subsidy on higher education loans, basic educational opportunity grants, and Job Corps payments. [10] Consists largely of Bureau of Indian Affairs payments, education exchange payments, Alaska Permanent Fund dividend payments, compensation of survivors of public safety officers, compensation of victims of crime, disaster relief payments, compensation for Japanese internment, and other special payments to individuals.

Source: U.S. Bureau of Economic Analysis, "Regional Accounts Data, Annual State Personal Income," <http://www.bea.gov/regional/spi>, accessed March 2011.
